# Explainable AI for Predicting Mortality Risk in Metastatic Cancer: Retrospective Cohort Study Using the Memorial Sloan Kettering-Metastatic Dataset

**DOI:** 10.2196/74196

**Published:** 2026-01-13

**Authors:** Polycarp Nalela, Deepthi Rao, Praveen Rao

**Affiliations:** 1 The University of Missouri Columbia, MO United States

**Keywords:** explainable artificial intelligence, machine learning in oncology, metastatic cancer, survivability prediction

## Abstract

**Background:**

Metastatic cancer remains one of the leading causes of cancer-related mortality worldwide. Yet, the prediction of survivability in this population remains limited by heterogeneous clinical presentations and high-dimensional molecular features. Advances in machine learning (ML) provide an opportunity to integrate diverse patient- and tumor-level factors into explainable predictive ML models. Leveraging large real-world datasets and modern ML techniques can enable improved risk stratification and precision oncology.

**Objective:**

This study aimed to develop and interpret ML models for predicting overall survival in patients with metastatic cancer using the Memorial Sloan Kettering-Metastatic (MSK-MET) dataset and to identify key prognostic biomarkers through explainable artificial intelligence techniques.

**Methods:**

We performed a retrospective analysis of the MSK-MET cohort, comprising 25,775 patients across 27 tumor types. After data cleaning and balancing, 20,338 patients were included. Overall survival was defined as deceased versus living at last follow-up. Five classifiers (extreme gradient boosting [XGBoost], logistic regression, random forest, decision tree, and naive Bayes) were trained using an 80/20 stratified split and optimized via grid search with 5-fold cross-validation. Model performance was assessed using accuracy, area under the curve (AUC), precision, recall, and *F*_1_-score. Model explainability was achieved using Shapley additive explanations (SHAP). Survival analyses included Kaplan-Meier estimates, Cox proportional hazards models, and an XGBoost-Cox model for time-to-event prediction. The positive predictive value and negative predictive value were calculated at the Youden index–optimal threshold.

**Results:**

XGBoost achieved the highest performance (accuracy=0.74; AUC=0.82), outperforming other classifiers. In survival analyses, the XGBoost-Cox model with a concordance index (C-index) of 0.70 exceeded the traditional Cox model (C-index=0.66). SHAP analysis and Cox models consistently identified metastatic site count, tumor mutational burden, fraction of genome altered, and the presence of distant liver and bone metastases as among the strongest prognostic factors, a pattern that held at both the pan-cancer level and recurrently across cancer-specific models. At the cancer-specific level, performance varied; prostate cancer achieved the highest predictive accuracy (AUC=0.88), while pancreatic cancer was notably more challenging (AUC=0.68). Kaplan-Meier analyses demonstrated marked survival separation between patients with and without metastases (80-month survival: approximately 0.80 vs 0.30). At the Youden-optimal threshold, positive predictive value and negative predictive value were approximately 70% and 80%, respectively, supporting clinical use for risk stratification.

**Conclusions:**

Explainable ML models, particularly XGBoost combined with SHAP, can strongly predict survivability in metastatic cancers while highlighting clinically meaningful features. These findings support the use of ML-based tools for patient counseling, treatment planning, and integration into precision oncology workflows. Future work should include external validation on independent cohorts, integration with electronic health records via Fast Healthcare Interoperability Resources–based dashboards, and prospective clinician-in-the-loop evaluation to assess real-world use.

## Introduction

Cancer remains one of the foremost global health challenges, with 611,720 deaths projected in the United States alone for 2024, and metastatic disease accounting for the overwhelming majority of these fatalities [1]. Metastasis is particularly vexing because disseminated tumor cells frequently acquire distinct genomic and phenotypic profiles that render them resistant to standard therapies. Contemporary oncology research, therefore, acknowledges intra- and intertumor heterogeneity as fundamental obstacles to curative treatment, therapeutic resistance, and accurate prognosis [2,3]. Reliable, early-stage prediction of patient survivability is pivotal not merely for counseling patients but also for tailoring aggressive interventions, prioritizing scarce health care resources, and designing adaptive clinical trials aimed at improving long-term outcomes.

Historically, clinicians have relied on the tumor-node-metastasis staging system, Kaplan-Meier life tables, and the Cox proportional hazards (CPH) regression model to stratify survival risk. Although statistically rigorous, these techniques impose proportional hazards and linearity assumptions that rarely hold across the dynamic, nonlinear biology of metastatic cancers. When violated, CPH models can yield biased hazard ratios, suffer from time-dependent covariate effects, and perform poorly on censored or highly imbalanced datasets [4].

The emergence of artificial intelligence (AI) and machine learning (ML) has enabled the development of sophisticated models that can uncover previously hidden patterns in heterogeneous clinical and multiomics datasets, thereby providing new insights into cancer biology, diagnosis, prognosis, and treatment outcomes. Over the past decade, ML models have repeatedly outperformed traditional statistical approaches. A deep-learning algorithm developed by Esteva et al [5] achieved a sensitivity of 97% and a specificity of 78% in classifying skin lesions as benign or malignant, while Liu et al [6] reported an area under the receiver operating characteristic curve (AUROC) of 0.94 for lung cancer risk prediction using computed tomography images. Similar gains have been demonstrated for breast cancer survival [7], lymph node metastasis [8], colorectal and soft tissue sarcoma outcomes [9], lung cancer survival [10], and prostate cancer prognosis [11,12].

Among contemporary ML pipelines, gradient-boosted decision trees, particularly extreme gradient boosting (XGBoost) [13], have emerged as a workhorse because they natively handle missing data, nonlinear feature interactions, and mixed data types. Recent examples include a non–small cell lung cancer microwave-ablation study where XGBoost achieved an area under the curve (AUC) of 0.89 [14]; a 2025 *Scientific Reports* analysis integrating survival models for breast cancer recurrence (concordance index [C-index]=0.82) [15]; a large colorectal cancer cohort where boosted trees yielded the highest 5-year survival accuracy [16]; and a thyroid cancer study that constructed a 10-year overall survival nomogram using Surveillance, Epidemiology, and End Results data [17].

Biomarkers are critical for early detection, diagnosis, prognosis, and monitoring. Traditional biomarker-discovery approaches often suffer from low sensitivity, limited reproducibility, and dependence on prior biological hypotheses. ML circumvents many of these limitations by integrating diverse data types and identifying complex nonlinear relationships. Algorithms such as random forests [18], support vector machines [19], and neural networks [20] have successfully identified biomarkers from gene expression [21-23], microRNA expression [24-27], DNA methylation [28,29], and imaging modalities [30-33].

Despite this progress, significant gaps persist at the intersection of scale, interpretability, and clinical use. First, while large datasets like the Memorial Sloan Kettering-Metastatic (MSK-MET) cohort [34] provide unprecedented scale, their analysis has largely relied on traditional statistics, failing to harness state-of-the-art ML for predictive modeling. Second, most ML survival-prediction studies focus on single tumor types, use modest sample sizes, or omit high-dimensional genomic features, limiting their generalizability to the pan-cancer reality of metastatic disease. Third, and most critically, interpretability remains a bottleneck; oncologists are understandably reluctant to incorporate opaque “black box” risk scores into clinical workflows. Explainable AI methods like Shapley additive explanations (SHAP) provide a mechanism for transparency. For instance, SHAP has revealed previously unknown drivers of prostate cancer mortality [35]. Although explainable AI frameworks offer a solution, large-scale, pan-cancer implementations that jointly optimize predictive performance and model explainability remain scarce. Consequently, the field lacks an interpretable, cross-tumor framework capable of ranking metastasis-specific risk factors at a scale that reflects real-world heterogeneity.

To address these critical gaps, we designed a methodological framework that moves beyond standard, single-model architectures. We leverage the MSK-MET dataset, a pan-cancer cohort of genomic and clinical data from 25,775 patients spanning 27 tumor types, as an ideal test bed for this purpose due to its scale and diversity. Our approach is conceived as a hierarchical and explainable pipeline specifically to tackle the challenges of data heterogeneity, the need for clinical trust, and biological discovery. It integrates rigorous pan-cancer benchmarking with targeted, tumor-specific submodels and crucially unifies ML classification with traditional survival analysis. This ensures that the predictive performance of our models is directly coupled with transparent, clinically actionable insights.

Driven by this methodology, our work addresses persistent gaps at the intersection of scale, interpretability, and clinical use in metastatic cancer prognosis. Leveraging a large pan-cancer cohort that captures real-world heterogeneity, we focus on whether explainable ML approaches can yield clinically useful survivability predictions while providing transparent, biologically and clinically coherent insights across tumor types.

The primary aim of this study is to develop and validate an interpretable ML framework for predicting overall survival in patients with metastatic cancer. We hypothesize that (1) ML models will achieve clinically useful discrimination and calibration for survivability prediction, (2) explainable AI techniques will identify a core set of prognostic biomarkers that are consistently important across diverse tumor types, and (3) integrating ML predictions with established survival analysis techniques will yield a transparent and clinically actionable tool for risk stratification.

## Methods

### Overview

**Figure 1:**
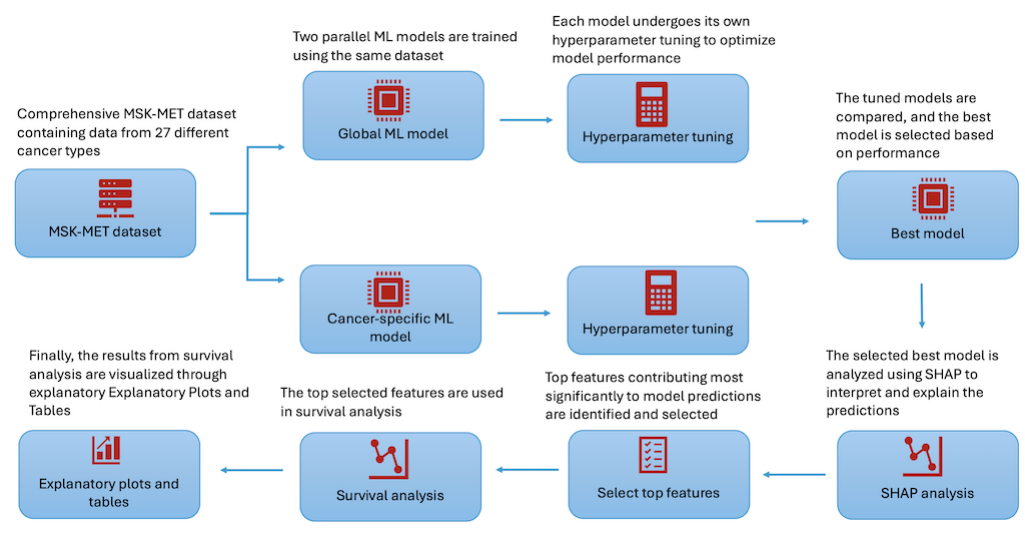
Overview of the explainable machine learning (ML) pipeline for metastatic cancer survivability prediction. The Memorial Sloan Kettering-Metastatic (MSK-MET) cohort is preprocessed and balanced, then split into stratified training and test sets before training and tuning 5 candidate classifiers (extreme gradient boosting [XGBoost], random forest, logistic regression, decision tree, and naive Bayes). The best-performing XGBoost model is subsequently interrogated with Shapley additive explanations (SHAP) to identify key prognostic clinical and genomic features, which are then carried forward into downstream survival analyses (Kaplan-Meier curves, Cox proportional hazards models, and XGBoost-Cox) to generate time-to-event estimates and clinically interpretable risk stratification.

Figure 1 illustrates the steps followed to predict cancer survivability using explainable AI. Raw data were used to initially train the ML models, followed by SHAP analysis. Top features identified by SHAP were then further used in the survival analysis. The subsequent steps below detail how this was implemented. All analysis and visualization were carried out in Python (version 3.12; Python Software Foundation) with relevant packages and libraries such as *pandas*, *numpy*, *scikit-learn*, *shap,* etc.

### Data Preprocessing

We first performed a thorough exploratory data analysis on MSK-MET that contained information from 25,775 patients with cancer. Our exploratory data analysis process began with a comprehensive analysis of the dataset, including the distribution of different cancer types, stages, and other relevant features. This helped us gain a deeper understanding of the underlying patterns and structures, which informed subsequent preprocessing steps. We preprocessed the input dataset and dropped columns (such as patient ID) and rows having large proportions of missing data. The target variable had 2 classes, that is, living (positive class, coded as 1) and deceased (negative class, coded as 0). In the final stage of preprocessing, the data were balanced using the target classes. Using resampling, we down-sampled the minority class (0). The final set contained 20,338 patients (10,169 living and 10,169 deceased) with 39 variables for each patient. In total, there were 27 cancer types (Table 1). The overall survival status was the target variable for prediction. Categorical variables were encoded using label encoding, and features were scaled using minimum-maximum scaling to ensure that variables with larger magnitudes did not unduly influence model outcomes. The resulting preprocessed data were then split into training and testing sets for further analysis.

**Table 1:** Distribution of primary cancer types in the Memorial Sloan Kettering-Metastatic cohort. Frequency counts are reported for all cancer types represented in the dataset; the 5 largest groups (non–small cell lung, colorectal, breast, pancreatic, and prostate cancer) provided the primary strata for cancer-specific extreme gradient boosting models and downstream survival analyses.

Cancer type	Frequency count, n
Non–small cell lung cancer	3790
Colorectal cancer	2696
Breast cancer	2043
Pancreatic cancer	1738
Prostate cancer	1596
Endometrial cancer	988
Ovarian cancer	923
Melanoma	882
Bladder cancer	870
Hepatobiliary cancer	790
Esophagogastric cancer	738
Soft tissue sarcoma	420
Head and neck cancer	362
Thyroid cancer	319
Renal cell carcinoma	318
Gastrointestinal stromal tumor	286
Small cell lung cancer	277
Germ cell tumor	241
Mesothelioma	219
Appendiceal cancer	160
Uterine sarcoma	133
Salivary gland cancer	123
Gastrointestinal neuroendocrine tumor	115
Skin cancer (nonmelanoma)	87
Cervical cancer	80
Small bowel cancer	76
Anal cancer	68

### Stratified Random Sampling of Training and Testing Sets

We used a stratified random sampling approach to create the training and test sets. First, we randomized the complete dataset to eliminate any inherent order or sequence. Then, we implemented stratification to ensure that the distribution of specific cancer types or stages in our training and testing sets mirrored that of the entire dataset. This is paramount to avoid potential biases and to ensure that our models have a representative sample of the different cancer types and stages present in the entire dataset. Following stratification, we allocated 80% of the data (16,270 patient records) to the training set while reserving the remaining 20% (4068 patient records) for the test set. This approach provides a robust foundation for model development and validation, ensuring both broad and deep representation of the dataset in our training and testing phases.

### Selection and Screening of ML Models for Cancer Survival Prediction

This study used 5 ML algorithms—XGBoost, naive Bayes, decision tree, logistic regression, and random forest—to predict cancer survival rates using the MSK-MET dataset. XGBoost was selected for its efficiency in handling sparse data and combining models to improve accuracy through ensemble learning. Naive Bayes, a simple classifier applying Bayes theorem, was chosen for its efficiency in high-dimensional datasets. The decision tree, known for its easy visualization and handling of nonlinear relationships, was included for its interpretability. Logistic regression was used for binary classification, predicting survival probabilities, while random forest, an ensemble method using multiple decision trees, was chosen for its accuracy and control over overfitting in large datasets.

### Hyperparameter Optimization Via Grid Search and Model Training

Grid search with hyperparameter tuning was applied to all 5 ML models. For XGBoost, parameters “n_estimators” (50-1000), “max_depth” (1-10), and “learning_rate” (0.01-0.3) were adjusted to optimize the number of trees, tree depth, and learning speed. Naive Bayes was tuned by varying “alpha” (0.01-10.0), “binarize” (0.0, 0.5, 1.0), and “fit_prior” (True/False). The decision tree’s grid search adjusted “max_depth” (None-10), “min_samples_split” (2, 5, 10), “min_samples_leaf” (1, 2, 4), and “criterion” (“gini” or “entropy”). Logistic regression was optimized with “C” (0.001-1000), “penalty” (“l1,” “l2,” “elasticnet,” “none”), and “solver” (“newton-cg,” “lbfgs,” “liblinear,” “sag,” “saga”). Random forest explored “n_estimators” (50, 100, 200) and “max_features” (“auto,” “sqrt”).

Using 5-fold cross-validation on the MSK-MET dataset, we trained the 5 classifiers to identify a robust, interpretable predictor and to derive a cohort-wide view of metastatic patterns.

### Model Evaluation

**Figure 2:**
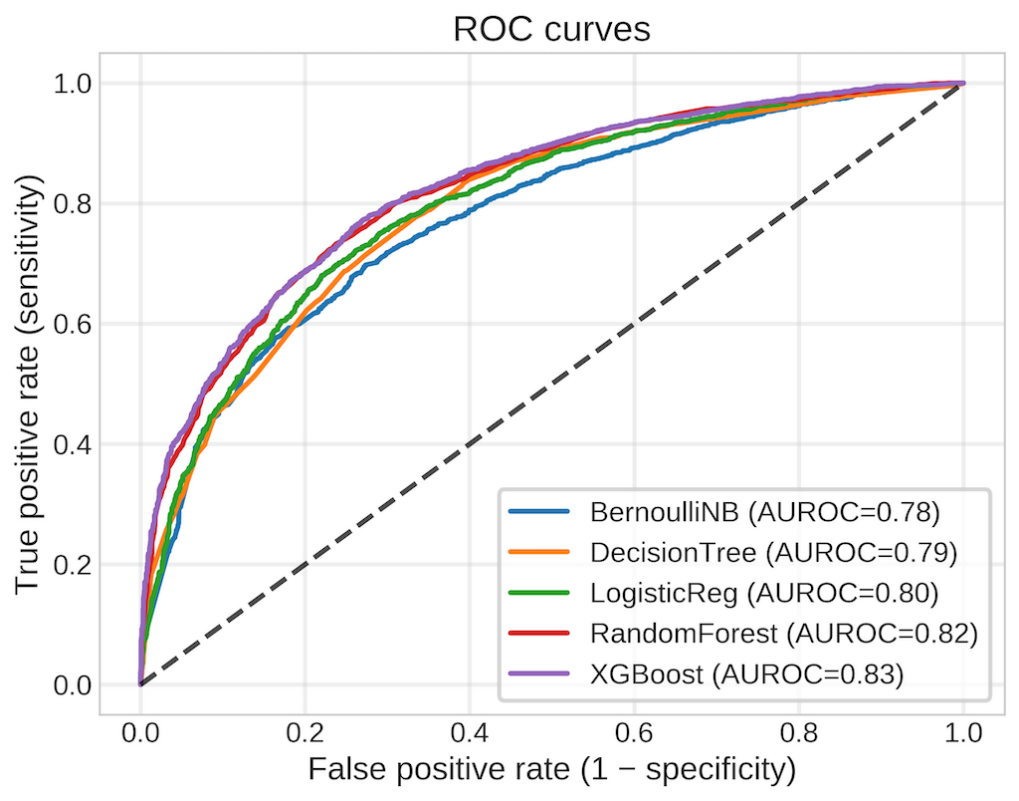
Receiver operating characteristic (ROC) curves for the 5 tuned classifiers on the held-out Memorial Sloan Kettering-Metastatic test set. Each curve shows the trade-off between sensitivity and 1 – specificity across decision thresholds, with corresponding area under the curve values summarized in Table 2. The extreme gradient boosting (XGBoost) model achieves the steepest ROC trajectory and highest area under the receiver operating characteristic curve (AUROC=0.82), indicating the strongest discrimination between surviving and deceased patients, while random forest and logistic regression (LogisticReg) form an intermediate tier, and decision tree and Bernoulli naive Bayes (BernoulliNB) exhibit comparatively weaker performance.

After training and testing the ML models on the MSK-MET dataset, we assessed their performance using 2 key metrics: the classification report (Table S2 in Multimedia Appendix 1) and AUROC (Figure 2). The AUROC measures the model’s ability to distinguish between classes, with higher AUC indicating better prediction. A score of 1 represents perfect predictions, 0.5 indicates random guessing, and below 0.5 suggests worse than random predictions. These metrics provide a comprehensive evaluation, ensuring the models not only predict accurately but also effectively identify positive cancer cases. This approach helps in selecting the best model for predicting cancer survival, balancing the need to detect true cases while minimizing false diagnoses.

To characterize operating-point behavior, we computed threshold-dependent trade-offs of true positive (TP), false positive (FP), true negative (TN), and false negative (FN); sensitivity, specificity, precision, negative predictive value (NPV), *F*_1_-score, balanced accuracy, Matthews correlation coefficient (MCC), Cohen κ, and accuracy at 3 decision thresholds: a fixed 0.50, the Youden J optimum, and a clinically constrained point targeting sensitivity ≥0.85. Scalar metrics were accompanied by 95% CI obtained via a 1000-sample stratified bootstrap.

To obtain unbiased estimates and avoid tuning leakage, we used nested repeated cross-validation on the training data. The outer loop applied RepeatedStratifiedKFold (5 folds × 10 repeats); within each outer training split, an inner 5-fold GridSearchCV tuned hyperparameters per model. Inner-tuned models were then scored on their corresponding outer validation splits, yielding a matrix of outer cross-validation (CV) results. The model with the highest mean outer-CV performance was refit on the full training set using the selected hyperparameters and evaluated once on the held-out test set (reporting accuracy, AUROC, and a confusion matrix).

For statistical comparison across the 5 models, we ran a Friedman omnibus test on the outer-CV score matrix. As a prespecified confirmatory analysis, we tested the directional hypothesis that random forest ≥ each baseline using 1-sided Wilcoxon signed-rank tests with Holm correction; we also reported effect sizes (mean and median paired differences) with 95% paired bootstrap CI. For rank-based visualization and exploratory post hoc inference, we computed average ranks with a Nemenyi critical-difference diagram and performed 1-sided Wilcoxon tests comparing the top-ranked model to the remaining models (Holm-adjusted). Where paired per-example test predictions were available, we used the McNemar test to compare error patterns between 2 models.

### Cancer Survival Prediction With XGBoost

XGBoost was therefore chosen for the rest of the analyses, involving both pan-cancer and cancer-specific models, metastatic cancer survival prediction, and survival analysis. To enhance transparency and clinical interpretability, we adopted a 2-tier XGBoost design. First, a unified XGBoost model was trained without the “Cancer Type” variable, leveraging clinical and demographic features to capture signals that generalize across diseases. Second, we trained cancer-specific XGBoost models for the 5 largest cancer groups identified in the cohort to capture within-disease interactions that can be diluted in a single global model. This combination provides both a cross-cancer perspective and disease-tailored insights that reflect differences in metastatic behavior and treatment context. In the final evaluation for both global and cancer-specific analysis, we focused only on measuring accuracy and AUC score.

### Model Interpretation and Explanation

To enhance the understandability and transparency of our predictions, we used XGBoost and SHAP for model explainability. SHAP, based on game theory, provides a detailed and consistent measure of feature importance by computing each feature’s contribution to the prediction. SHA*P* values represent a feature’s responsibility for a change in the model output, ensuring local accuracy, missingness, and consistency. This method quantifies the impact of each feature on predictions and explains how the presence or absence of a feature affects the outcome. In our SHAP analysis, survival is the positive class. Beeswarm plots are particularly useful for visualizing SHA*P* values, showing features’ influence and variability in a nuanced manner.

### Survival Analysis

Following the training of the XGBoost ML model and SHAP analysis, the most important features, such as metastatic site count, tumor mutational burden (TMB), and specific organ metastases (eg, liver, bone, and lung) influencing patient survival, were identified and used in the survival analysis. The primary goal was to examine the duration from cancer diagnosis to patient death, assessing how clinical and genomic variables impact survival times. We used Kaplan-Meier survival analysis, CPH modeling, log-rank tests for comparing survival distributions, and XGBoost survival analysis to deepen our understanding of patient outcomes. All of the original data was used in the survival analysis.

### Kaplan-Meier Survival Analysis

The Kaplan-Meier estimator was used to evaluate survival probabilities over time across different patient subgroups. Patients were stratified based on key features identified from SHAP analysis. Survival curves were compared using the log-rank test to assess statistically significant differences between groups. A *P* value of <.05 was considered statistically significant. The analysis was first performed on all the data to assess survival of patients with metastatic versus nonmetastatic disease and then on subgroups of the top 5 cancer types including non–small cell lung cancer, colorectal cancer, breast cancer, pancreatic cancer, and prostate cancer.

### CPH Model

The CPH model was applied to assess the influence of multiple covariates on patient survival while controlling for potential confounders. Key covariates included metastatic site count, fraction of genome altered, TMB, and distant metastases in specific organs. The proportional hazards assumption was evaluated using Schoenfeld residuals, and any violations were addressed through stratification or inclusion of time-varying covariates. Hazard ratios with corresponding 95% CI were reported to quantify risk associations.

### Log-Rank Test

To further compare survival distributions between different patient cohorts, the log-rank test was applied. This test was used to determine whether survival differences observed between patient subgroups (eg, metastatic vs nonmetastatic) were statistically significant. The resulting *P* values guided the identification of meaningful clinical predictors. Furthermore, a plot was generated for the Kaplan-Meier survival curves with the overall survival (months) on the x-axis and survival probability on the y-axis.

### XGBoost Survival Analysis

To capture complex, nonlinear relationships and interactions among variables, XGBoost survival analysis was implemented. This adaptation of XGBoost used a Cox-based loss function to accommodate censored survival data. Hyperparameter tuning was conducted using grid search, optimizing parameters such as “n_estimators,” “max_depth,” and “learning_rate.” The model’s C-index was used to evaluate predictive performance. SHA*P* values were also applied to the survival model to interpret feature importance and explore individual risk predictions.

### Ethical Considerations

This study analyzed secondary, noninterventional data from the MSK-MET cohort obtained via a publicly accessible repository (cBioPortal for Cancer Genomics). All records used for modeling and statistical analysis were anonymous and deidentified prior to access; no direct identifiers (eg, names, street addresses, full dates of birth, medical record numbers) or indirect reidentification keys were available to the research team. Because only deidentified data were used and no contact with human participants occurred, the work was considered non–human participants research and did not require informed consent or additional institutional review.

Data handling procedures followed best-practice privacy safeguards. Working datasets were stored on access-controlled systems. We did not attempt any record linkage or reidentification. To support responsible AI, model development incorporated transparent methods (eg, SHAP explanations) and prespecified subgroup evaluations to screen for potential performance disparities. All code and evaluation protocols are shared to enable reproducibility without exposing any protected information.

## Results

### Model Performance

The evaluation of the 5 distinct models on the MSK-MET dataset yielded a spectrum of performances. The overall classification performance for the 5 classifiers is summarized in Table 2. Receiver operating characteristic (ROC) curves are shown in Figure 2, and precision-recall curves are shown in Figure S2A, calibration in Figure S2B, decision-curve analysis in Figure S2C, and threshold-dependent metrics in Figure S2D in Multimedia Appendix 1.

**Table 2:** Discrimination and calibration of tuned classifiers on the same test set. Reported are area under the curve (AUC), area under the precision-recall curve (AUPRC), and Brier score, each with 95% CIs; the outcome prevalence was 0.50. Extreme gradient boosting (XGBoost; lr=0.01; depth=5; n=500) showed the strongest overall performance, with the highest AUC and AUPRC and the lowest Brier score, followed by random forest, while logistic regression, decision tree, and naive Bayes exhibited progressively lower discrimination and less favorable calibration.

Model	AUC (95% CI)	AUPRC (95% CI)	Brier score (95% CI)
XGBoost (lr=0.01; depth=5; n=500)	0.82 (0.81-0.84)	0.83 (0.81-0.85)	0.17 (0.16-0.18)
Random forest (n_estimators=200; max_features=auto)	0.80 (0.78-0.81)	0.81 (0.79-0.82)	0.18 (0.18-0.19)
Logistic regression (C=10; l2; liblinear)	0.79 (0.78-0.81)	0.79 (0.77-0.81)	0.19 (0.18-0.19)
Decision tree (gini; depth=8; min_leaf=1; min_split=10)	0.78 (0.77-0.80)	0.78 (0.76-0.80)	0.19 (0.18-0.20)
Naive Bayes (α=10.0; binarize=0.5; fit_prior=False)	0.78 (0.77-0.80)	0.77 (0.75-0.79)	0.21 (0.20-0.22)

XGBoost demonstrated the strongest discrimination and probability quality. It achieved an AUC of 0.82 (95% bootstrap CI 0.81-0.84) and the highest area under the precision-recall curve (AUPRC) at approximately 0.83 against a baseline precision equal to the prevalence (0.50; Table 2 and Table S2 in Multimedia Appendix 1). In accuracy terms, XGBoost reached 0.74 (3010/4068) and, for our summary counts, corresponded to 3335 out of 4068 test patients correctly stratified at the chosen operating point. Random forest (AUC=0.80; AUPRC=0.81) and logistic regression (AUC=0.79; AUPRC=0.79; accuracy=0.72; 2929/4068) formed a consistent middle tier, while decision tree and Bernoulli naive Bayes trailed slightly (both AUC=0.78; accuracy=0.72; 2929/4068; correctly stratified=3173/4068 for AUC-aligned counts; see Table 3 and Table S3 in Multimedia Appendix 1 for detailed counts and metrics). These rankings were concordant across ROC and precision-recall analyses and remained stable across threshold-dependent operating points (fixed threshold=0.50, Youden J optimum, and a clinically constrained sensitivity ≥0.85; Figure 2; Figures S2A and S2D and Tables S2 and S3 in Multimedia Appendix 1).

Calibration analyses reinforced this ordering. XGBoost produced the lowest Brier score (approximately 0.17; Table 2; Table S2 and Figure S2B in Multimedia Appendix 1) and a reliability curve that closely tracked the 45° line, with logistic regression comparably well-calibrated, whereas Bernoulli naive Bayes deviated most at the extremes (Table 2; Table S2 and Figure S2B in Multimedia Appendix 1).

**Table 3:** Compact comparison of 5 classifiers across 3 operating points, that is, default probability cutoff (0.50), Youden J (threshold maximizing sensitivity + specificity – 1), and a high-sensitivity setting (constrained to sensitivity ≥0.85). For each model-threshold pair, we report sensitivity, specificity, Matthews correlation coefficient (MCC), and accuracy; thresholds are applied to the predicted positive-class probability. This summary emphasizes decision-relevant trade-offs: balanced performance at Youden J and the specificity cost of prioritizing high sensitivity. In this cohort, extreme gradient boosting (XGBoost) yields the strongest balanced performance (highest MCC and accuracy) at Youden J while retaining the best specificity among the high-sensitivity operating points.

Model and operating point		Threshold		Sensitivity		Specificity		MCC		Accuracy
**Naive Bayes**										
	Probability cutoff 0.5	0.50	0.66		0.78		0.44		0.72	
	Youden J	0.50	0.65		0.78		0.44		0.72	
	Sensitivity ≥0.85	0.15	0.85		0.52		0.39		0.69	
**Decision tree**										
	Probability cutoff 0.5	0.50	0.72		0.71		0.43		0.72	
	Youden J	0.56	0.64		0.80		0.45		0.72	
	Sensitivity ≥0.85	0.32	0.86		0.49		0.37		0.67	
**Logistic regression**										
	Probability cutoff 0.5	0.50	0.73		0.73		0.45		0.73	
	Youden J	0.53	0.69		0.77		0.46		0.73	
	Sensitivity ≥0.85	0.38	0.85		0.54		0.41		0.69	
**Random forest**										
	Probability cutoff 0.5	0.50	0.69		0.76		0.45		0.72	
	Youden J	0.49	0.70		0.75		0.46		0.73	
	Sensitivity ≥0.85	0.32	0.85		0.54		0.41		0.69	
**XGBoost**										
	Probability cutoff 0.5	0.50	0.72		0.78		0.50		0.75	
	Youden J	0.50	0.72		0.78		0.50		0.75	
	Sensitivity ≥0.85	0.33	0.85		0.58		0.45		0.72	

Threshold-dependent performance, summarized from confusion matrix–derived metrics at 3 operating points, made the error trade-offs explicit (Table 2; Table S3 and Figure S2D in Multimedia Appendix 1). At a fixed 0.50 threshold essentially indistinguishable from the Youden J optimum on this balanced test set, XGBoost balanced sensitivity and specificity most effectively (sensitivity=0.72; specificity=0.78; positive predictive value (PPV)=0.77; NPV=0.74; *F*_1_-score=0.74; MCC=0.50; accuracy=0.75). Under a clinically constrained operating point prioritizing case-finding (sensitivity ≥0.85), XGBoost required a threshold of approximately 0.33 and achieved a sensitivity of 0.85 (specificity=0.58; PPV=0.67; NPV=0.80; *F*_1_-score=0.75; MCC=0.45; accuracy=0.72). The corresponding counts at this setting were TP=1731, FP=846, TN=1188, and FN=303 (n=4068). Comparator models met the same sensitivity target with lower specificity and weaker composite indices; for example, random forest and logistic regression both settled near a specificity of 0.54 with MCC=0.41, while decision tree and Bernoulli naive Bayes lost additional specificity and MCC. These results indicate that, when sensitivity is held high, XGBoost preserves more TNs and maintains stronger global agreement (MCC, balanced accuracy).

Decision-curve analysis supported the same ordering of clinical use across a broad range of threshold probabilities (approximately 0.15-0.70), with XGBoost yielding the highest net benefit, random forest next, and logistic regression close behind (Figure S2C and Table S2 in Multimedia Appendix 1). Together with the calibration findings, these analyses suggest that XGBoost not only separates cases from controls most effectively but also produces usable risk estimates for thresholding and shared decision-making.

Furthermore, when the global XGBoost model was compared to cancer-specific XGBoost models for the top 5 cancer types (non–small cell lung, colorectal, breast, pancreatic, and prostate cancer; Tables 4 and 5), the prostate cancer model emerged as the most accurate, with an AUC of 0.88 and an accuracy of 0.84. (268/319 test patients) and an AUC of 0.88 (281/319 test patients correctly stratified). Meanwhile, pancreatic cancer posted a lower AUC of 0.68 (236/348 test patients correctly stratified), reflecting greater challenges in classification for that subgroup.

**Table 4:** Condensed Cox proportional hazards model for overall survival in the Memorial Sloan Kettering-Metastatic cohort. Hazard ratios (HRs), 95% CIs, and *P* values are reported for the most influential covariates identified in the global model, showing that metastatic status, higher metastatic site count, increased tumor mutational burden (TMB), greater fraction of genome altered, and distant metastases to liver, bone, and lung are all associated with significantly elevated mortality risk, whereas primary sample type is modestly protective.

Risk factor	HR (95% CI)	*P* value
Patient with metastatic disease	2.18 (1.97-2.42)	*<.*001
Metastatic site count	1.03 (1.02-1.04)	*<.*001
TMB (nonsynonymous)	1.00 (0.99-1.00)	*<.*001
Fraction of genome altered	1.32 (1.19-1.46)	*<.*001
Sample type (primary=ref)	0.87 (0.83-0.90)	*<.*001
Distant metastasis: liver	1.81 (1.73-1.90)	*<.*001
Distant metastasis: bone	1.43 (1.37-1.50)	*<.*001
Distant metastasis: lung	1.16 (1.11-1.22)	*<.*001

**Table 5:** Comparison of classification performance between the unified or global extreme gradient boosting (XGBoost) model and cancer-specific XGBoost models. Overall accuracy and area under the curve (AUC) are reported for each classifier, showing that while the global model achieves strong baseline discrimination (accuracy=0.74; AUC=0.82), several cancer-specific models, particularly prostate and breast cancer, attain even higher AUCs (0.88 and 0.85, respectively), whereas the pancreatic cancer model lags behind, reflecting underlying heterogeneity in predictability across tumor types.

Classifier	Accuracy score	AUC score
Unified or global model	0.74	0.82
Non–small cell lung model	0.71	0.79
Colorectal cancer model	0.73	0.81
Breast cancer model	0.76	0.85
Pancreatic cancer model	0.72	0.68
Prostate cancer model	0.84	0.88

### Model Explainability

**Figure 3:**
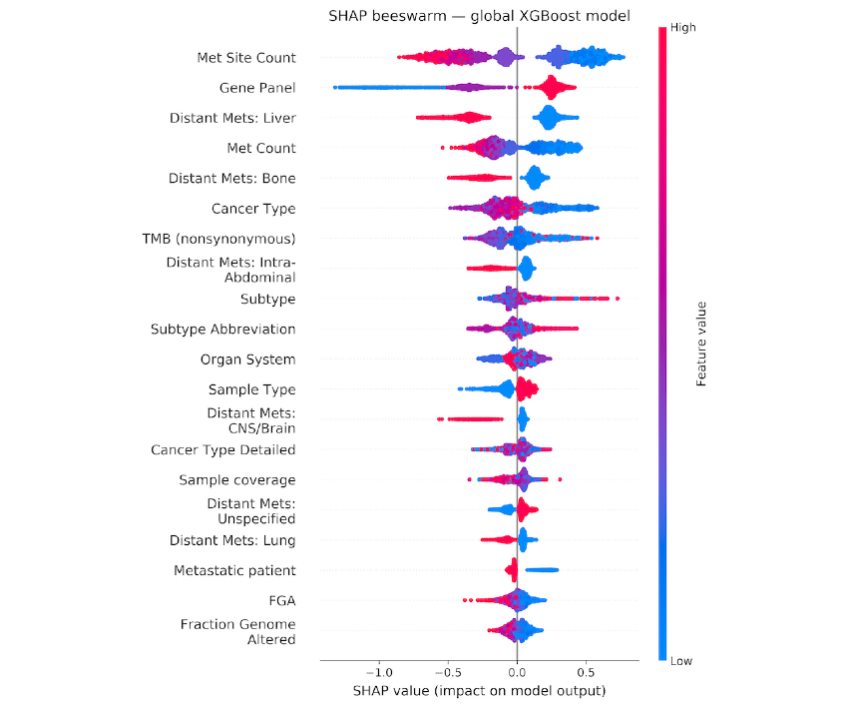
Shapley additive explanations (SHAP) beeswarm plot for the global extreme gradient boosting (XGBoost) mortality classifier in the Memorial Sloan Kettering-Metastatic cohort. Features are ordered by mean absolute SHAP value, highlighting metastatic (Met) site count, tumor mutational burden (TMB), fraction of genome altered (FGA), and distant metastases to the liver and bone as the strongest drivers of model predictions. Each point represents a patient, with horizontal position indicating the direction and magnitude of impact on predicted mortality risk and color denoting low (blue) to high (red) feature values, illustrating how extreme values systematically shift risk estimates. Central nervous system is denoted by CNS.

The SHAP analysis of the global XGBoost model established a clear hierarchy of feature importance, identifying metastatic site count, TMB, fraction of genome altered, and distant metastases to the liver and bone as the predominant prognostic factors (Figure 3 and Table S6 in Multimedia Appendix 1). The beeswarm plots for these top features demonstrated a robust predictive power, with high and low feature values cleanly separating along the SHA*P* value axis, indicating a consistent and strong directional impact on model output (Figures S3A-S3E in Multimedia Appendix 1).

This analysis was extended to cancer-specific models, revealing a critical dual perspective, that is, the core features identified in the global model recurrently ranked among the most important across individual cancer types, while disease-specific features also emerged. For instance (Table S6 and Figures S3A-S3E in Multimedia Appendix 1), the global top features like metastatic burden and liver metastases remained highly influential in specific models such as colorectal and prostate cancer. Concurrently, the models identified context-specific predictors, such as distant metastasis in the lung for non–small cell lung cancer, sample type for breast cancer, and distant metastasis in the male genital for prostate cancer (Figure 3; Figures S3D and S3E in Multimedia Appendix 1). This underscores that while a common set of pan-cancer drivers exists, the models successfully capture nuanced, disease-specific metastatic behaviors.

**Figure 4:**
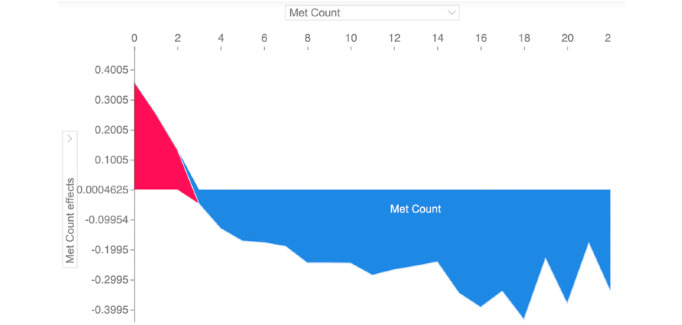
Shapley additive explanations force plot illustrating the impact of metastatic (Met) site count on predicted mortality risk for the first 1000 patients in the Memorial Sloan Kettering-Metastatic test set. Each horizontal bar represents an individual patient, with the baseline prediction shown at the center and shifts toward higher (right, red) or lower (left, blue) mortality risk driven by the number of metastatic sites. The consistent rightward push associated with increasing metastatic site count visually confirms its strong, monotonic contribution to higher predicted risk, reinforcing the biological and clinical plausibility of the extreme gradient boosting model’s behavior.

The consistent importance of features like metastatic site count was further validated by the SHAP force plot (Figure 4), which visually confirmed that an increasing number of metastatic sites directly correlated with a higher model output for mortality risk, reinforcing the clinical and biological plausibility of the model’s predictions.

### Survival Analysis

**Figure 5:**
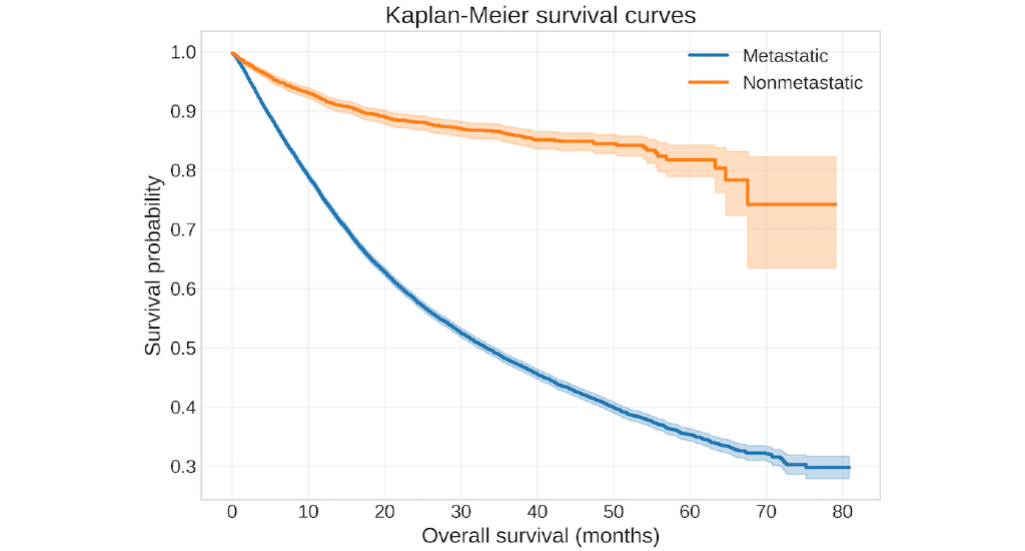
Kaplan-Meier survival curves comparing overall survival in patients with vs without metastatic disease in the Memorial Sloan Kettering-Metastatic cohort. Patients with metastases demonstrate markedly lower survival probabilities and earlier median survival times than patients without metastases, underscoring the substantial impact of metastatic status on long-term outcomes.

In the Kaplan-Meier analysis (Figure 5 and Table S7 in Multimedia Appendix 1), patients were stratified into “metastatic” and “nonmetastatic” groups to compare differences in overall survival. The survival probability of patients in the metastatic group was notably lower than that of the nonmetastatic group, as seen in the pronounced separation of their survival curves. By approximately 80 months, patients with metastatic disease exhibited a survival probability of 0.30 (3041/10*,*169 patients with metastatic disease) versus 0.80 (8135/10*,*169 patients without metastatic disease), underscoring the substantial impact of metastatic status on long-term survival outcomes.

Subgroup survival results for the top 5 cancer types were as follows: in non–small cell lung cancer (n=4686), 43.75% (n=2050) of patients experienced an event and 82.5% (n=3866) presented with metastatic disease; the median overall survival was 17.7 (IQR 8.4-33.2) months. In colorectal cancer (n=3541), the event rate was 33.52% (n=1187), the metastatic proportion was 90.82% (n=3216), and the median survival was 17.5 (IQR 10-30) months.

In breast cancer (n=2601), 35.64% (n=927) experienced an event and 76.47% (n=1989) had metastatic disease, with a median survival of 33 (IQR 14.4-43.4) months. In pancreatic cancer (n=1981), the event rate was 60.27% (n=1194), the metastatic proportion was 93.54% (n=1853), and the median survival was 11.6 (IQR 4.2-21.8) months. Finally, in prostate cancer (n=2166), 26.45% (n=573) experienced an event and 81.12% (n=1757) had metastatic disease, with a median survival of 21.6 (IQR 11.6-38.2) months (Figure S5A-S5E in Multimedia Appendix 1).

In the CPH model (Table 4), factors like metastatic site count, TMB, fraction of genome altered, and distant metastases displayed hazard ratios above 1.0, indicating an increased risk of mortality. These relationships attained statistical significance, with *P* values under the established threshold. The proportional hazards assumption was checked through Schoenfeld residuals, and only minor deviations were noted, which did not substantially affect the covariate estimates. The model’s C-index reached approximately 0.66, reflecting moderate predictive power in distinguishing survival outcomes among different patient subgroups.

An XGBoost survival model, fitted with a Cox-based loss function, achieved a higher C-index (0.7) than the standard Cox model. In Table S7 in Multimedia Appendix 1, the model’s important features are displayed, with distant metastasis, TMB, and fraction of genome altered, among others, listed as the most important features that influence the prediction.

## Discussion

### Principal Findings

Using a large, multitumor metastatic cohort (MSK-MET), we developed explainable ML models for survivability prediction and complemented them with time-to-event modeling. Among conventional classifiers, XGBoost delivered the best overall performance, achieving an accuracy of 0.74 and AUC=0.82 on the held-out test set. In parallel, an XGBoost-Cox variant improved time-to-event concordance over a standard Cox model (C-index=0.70 vs 0.66, with key hazard ratios from the Cox model). Model explanations (SHAP) and hazard-based analyses converged on clinically recognizable prognostic factors: the number of metastatic sites, TMB, fraction of genome altered, and the presence of liver and bone metastases, while cancer-specific performance varied in biologically plausible ways (eg, higher AUC in prostate and lower in pancreatic). At the Youden-optimal threshold, PPV was approximately 70% and NPV approximately 80%, indicating practical use for risk stratification in balanced decision contexts. We expand the discussion of these findings in the following subsections.

### Model Performance, Robustness, and Benchmarking

Our evaluation moved beyond accuracy to provide a comprehensive assessment across discrimination, calibration, operating-point trade-offs, and clinical use. XGBoost consistently emerged as the most dependable model, with its incremental gains in AUC and AUPRC translating into more favorable confusion-matrix profiles at clinically relevant thresholds. For instance, when sensitivity is constrained to be high, XGBoost retains more specificity and a higher MCC, reducing FPs without sacrificing case finding. Its well-calibrated probability estimates are crucial for decision support, enabling rational threshold selection and clear communication of absolute risk.

The model’s practical use is further evidenced by its performance across different decision contexts. For balanced decision-making, a default threshold near 0.50 (coinciding with the Youden J optimum) provides a sensible starting point. In triage-like scenarios demanding high sensitivity, a lower threshold around 0.33 yields sensitivity near 0.85 with tolerable specificity losses and an NPV around 0.80. Decision-curve analysis confirmed that XGBoost provides a larger net benefit across a wide band of threshold probabilities, suggesting robustness to varying clinical preferences.

These performance results harmonize with model explanations and survival evidence. SHAP analyses validate that predictions are driven by biologically sensible covariates, while survival curves and hazard ratios show coherent, directionally consistent effects. This triangulation across discrimination, calibration, interpretability, and survival analysis adds credibility that the learned signal reflects underlying disease biology rather than being an artifact of the classifier or data split.

### Parameter Sensitivity and Model Robustness

During extensive grid-search tuning, we found that XGBoost hyperparameters, especially tree depth, learning rate, and number of estimators, greatly influenced AUC and classification accuracy. Shallow trees underfit, while deeper ones improved performance but risked overfitting in smaller cancer-specific cohorts. Learning rates below 0.05 caused slow convergence, while overly high rates destabilized training. Despite this variability, certain predictive features, particularly metastatic burden and genomic alterations, remained consistently impactful, underscoring the robustness of our model. Future research could explore adaptive optimization techniques such as Bayesian optimization or reinforcement learning for enhanced generalizability.

### Is the Performance “Good Enough”?

The discriminatory performance of prognostic models is central to their clinical use. In oncology, C-index or AUC of at least 0.70 is generally regarded as the minimum threshold for clinical usefulness, while values exceeding 0.80 are considered strong and often necessary for clinical translation [36,37]. These interpretive standards are rooted in established methodological work on ROC analysis, where AUC values between 0.7 and 0.8 are typically described as “acceptable” and those above 0.8 as “excellent” [36,37].

Evidence from systematic reviews of head and neck cancer prognostic models demonstrates how these thresholds translate into practice. Philip et al [38] reported that most radiomics-based prognostic models achieved C-indices below 0.70, underscoring their weak discriminatory power and limited clinical applicability. In contrast, externally validated models that approached or exceeded the 0.80 mark, as summarized by Dretzke et al [39], were identified as more robust and clinically relevant, supporting their potential integration into patient management. These findings emphasize that models below the 0.70 threshold may remain academically interesting but lack sufficient reliability for routine use, whereas those meeting or surpassing 0.80 provide stronger grounds for clinical adoption.

Comparisons with deep learning approaches further reinforce this interpretation. Gouthamchand et al [40] found that the most competitive deep learning models in head and neck oncology consistently achieved AUCs of 0.80 or higher, placing them above many handcrafted radiomics models. This pattern suggests that 0.80 represents not merely an aspirational benchmark but an increasingly practical requirement for prognostic tools aiming to match or surpass the performance of advanced ML methods already being piloted in clinical contexts.

Regulatory precedents also align with this threshold. Analyses of Food and Drug Administration (FDA)–cleared AI- and ML-enabled medical devices, such as the LumineticsCore software, indicate that most systems demonstrate discriminatory performance in the high 0.7 to 0.9 range, with pivotal studies for devices such as IDx-DR reporting AUCs above 0.80. Joshi et al [41] provide a comprehensive overview of this landscape, confirming that AUCs in this range are characteristic of AI systems deemed safe and effective for clinical use. FDA guidance similarly emphasizes the importance of demonstrating robust discriminatory performance in supporting claims of clinical effectiveness [42].

However, the thresholds discussed here should not be treated as rigid cutoffs. Clinical acceptability is not defined by a single number but by the context of the decision, the potential harms and benefits, and the baseline risk of the population. In high-stakes oncology decisions such as treatment intensification that carries significant morbidity, a well-calibrated model with an AUC of 0.78 but demonstrable net benefit at clinically relevant thresholds may be more valuable than a model with an AUC of 0.84 that is poorly calibrated or unstable. Thus, performance thresholds should be interpreted as contextual benchmarks rather than universal standards.

It is also important to recognize that discrimination does not equal usefulness. AUC or C-index quantifies a model’s ability to rank patients by risk, but it does not measure whether the absolute risk estimates are accurate. For clinical practice, calibration is equally critical whether a patient predicted to have a 30% risk actually experiences that outcome about 30% of the time. Calibration-in-the-large, calibration slope, Brier score, and visual calibration plots provide this essential information. Moreover, calibration at clinically meaningful cut points (eg, deciles of predicted risk) is necessary to ensure predictions are trustworthy for patient counseling and decision-making.

Finally, prognostic end points in oncology are often time-to-event outcomes. In such settings, the C-index is commonly used, but time-dependent AUCs provide more clinically interpretable information by specifying the prediction horizon (eg, 12-month or 36-month survival). This allows clinicians to understand how well the model performs over the actual time frames relevant to follow-up and treatment decisions. Where applicable, handling of censoring and competing risks should also be reported, particularly if the terminal outcome is mortality.

Taken together, these considerations emphasize that while an AUC ≥0.80 is encouraging and aligns with many published benchmarks, true clinical use depends on calibration, decision-curve analysis, and performance at specific decision thresholds relevant to patient care.

### Clinically Actionable Metrics, Implications, and Use

The performance metrics of our optimal XGBoost model are translated into clinically actionable insights through decision-curve analysis. At the threshold maximizing the Youden index, the model achieves a PPV of 70.3% and an NPV of 79.8%. This indicates that a high-risk prediction from the model would correspond to an actual terminal outcome in approximately 7 out of 10 cases, supporting its use in justifying intensified monitoring or treatment. Conversely, the high NPV means 8 out of 10 low-risk predictions correctly identify patients with a more favorable prognosis, providing a quantitative basis for discussions about de-escalating care and reducing treatment-related morbidity.

Critically, the decision-curve analysis confirms the model’s practical use across a spectrum of clinical decision-making preferences. The analysis demonstrates that the XGBoost model provides a superior net benefit compared to both alternative models and the default strategies of treating all or no patients across a wide range of threshold probabilities. This indicates that using the model to guide decisions is clinically advantageous regardless of whether the clinician prioritizes avoiding FPs (overtreatment) or FNs (missed interventions). The point at which the net benefit of the model crosses the “treat all” strategy is particularly important, as it defines the minimum probability at which the model’s prediction becomes more useful than intervening in every case. The sustained positive net benefit of our model underscores its robustness and potential to improve patient outcomes by aligning interventions with individualized risk.

The combination of strong predictive performance and model explainability supports several immediate clinical use cases: (1) patient triage and counseling via individualized risk summaries, (2) treatment planning and shared decision-making aided by feature-level rationales, and (3) integration into electronic health record (EHR)–embedded dashboards for longitudinal monitoring. The top predictive features are routinely available in most cancer centers, facilitating adoption with minimal workflow disruption.

### Model Explainability and Biological Plausibility

Our model selection was guided by metrics that reflect clinical reality, prioritizing both accuracy and AUROC. The AUROC is especially critical in medical settings, as it captures the essential balance between TPs and FPs, where the cost of FNs (missed cases) is high [43]. This evaluation consistently identified XGBoost as the top performer. Its superior ability to capture complex, nonlinear relationships within high-dimensional clinical and genomic data, as evidenced by its significant lead over other models, aligns with established literature on gradient boosting for cancer predictions [44,45]. The model’s discriminative power is substantial, with an AUC of 0.82 representing an excess over chance of 0.32. This can be intuitively communicated as a “number needed to screen” of approximately 3-4 patients to identify 1 additional correct classification relative to chance, providing a tangible sense of clinical yield. This strong performance is operationalized at the Youden-optimal threshold, where PPV of approximately 70% and NPV of approximately 80% offer concrete use for risk stratification, effectively ruling in or ruling out high-risk status to guide downstream clinical actions.

Crucially, this high performance is rendered transparent and trustworthy through SHAP-based interpretation. The model’s decisions are not black box outputs but are demonstrably driven by biologically plausible features that align with oncological principles. SHAP analysis consistently identified metastatic site count, liver and bone metastases, TMB, and fraction of genome altered as the top predictors; moreover, these findings are well-supported in the literature [46]. This biological plausibility was further refined and validated in our cancer-specific models. For example, the prominence of lung metastases in non–small cell lung cancer and prostate cancer models, and the major role of sample type in the breast cancer model, demonstrate a nuanced understanding of disease-specific pathophysiology as pointed out in other studies [47]. The distinct, right-skewed SHAP distribution for “metastatic count” and its direct correlation with risk in force plots further reinforced the importance of total lesion burden in driving high-risk predictions. This coherence between model explanations and established clinical knowledge across both pan-cancer and disease-specific contexts is fundamental for building clinician trust and facilitating the integration of this tool into point-of-care decision support.

### Survival Modeling and Risk Stratification

#### Time-to-Event Insights

Survival analysis confirmed classification results. Kaplan-Meier curves revealed steep survival drops in patients with versus without metastatic disease (0.3 vs 0.8 at 80 months). Traditional Cox models identified high hazard ratios for metastatic site count, TMB, and fraction of genome altered but struggled with their linearity assumptions (C-index=0.66). In contrast, XGBoost-based survival modeling better captured nonlinearity, achieving a higher C-index of 0.70.

#### Subgroup Survival Analysis

The 5 disease-specific results highlight substantial heterogeneity in survival outcomes across cancer types within the same analytic framework. Pancreatic cancer exhibited the shortest median survival alongside the highest event rate and metastatic proportion, underscoring its aggressive clinical course even within a predominantly metastatic cohort. By contrast, breast cancer showed the longest median survival (as visualized in the respective Kaplan-Meier curves) despite a sizable metastatic share, suggesting comparatively slower disease trajectories and greater effectiveness of available therapies in this subgroup. Non–small cell lung cancer and colorectal cancer shared similar median survivals near 17-18 months, though colorectal cancer carried the highest metastatic proportion among the five. This juxtaposition implies that crude metastatic prevalence alone does not fully account for survival differences, motivating disease-specific modeling of covariates and sites of spread. Prostate cancer combined the lowest event rate with a midrange metastatic proportion and an intermediate median survival, indicating a slower accumulation of events over time relative to the other cancers. Together, these patterns justify presenting separate Kaplan-Meier curves and parsimonious Cox summaries per cancer type, while enabling a consistent cross-cancer narrative that focuses on differences in event rates, metastatic burden, and median survival.

### Novelty and Comparison to Prior Work

Although significant studies [48-52] (Table S8 in Multimedia Appendix 1) have been conducted in the realm of predicting cancer survivability, our work stands out by bringing new enhancements that significantly contribute to better prediction of cancer survivability, particularly by the thorough comparison of ML models, the strategic use of both global and cancer-specific models, in-depth model explainability using SHA*P* values, and detailed survival analysis.

First, we begin by comparing 5 different ML models (XGBoost, naive Bayes, decision tree, logistic regression, and random forest), each rigorously tuned using exhaustive grid search for hyperparameters. This approach ensures that each model is thoroughly tested for the task of predicting cancer survivability, a detail that is often overlooked in existing literature. Many studies tend to focus on one or two models, without ensuring that the models are fully evaluated for comparison. For example, prior work done by Zhao et al [48], Tapak et al [49], and Nicolò et al [50] evaluate models but lack the thoroughness in hyperparameter tuning that our study provides. We believe that this rigorous approach enhances the reliability of our findings and provides a more comprehensive understanding of which model performs best under specific conditions.

Moreover, we use a methodology that is designed to first use a global model to gain a general overview of the most important patterns and predictors for metastatic cancer survivability, followed by a deeper dive into cancer-specific models. This 2-tiered approach is critical because it allows us to identify broad patterns while also uncovering nuances that might be missed or misinterpreted in a global model. Many published studies, such as Kourou et al [51] and Zhao et al [48], predominantly dwell on global accuracy metrics without taking this crucial next step to explore more specific patterns within subgroups of the data. By contrast, our approach provides a dual perspective, that is, broad insights from the global model and detailed, cancer-specific insights that we believe are essential for advancing personalized medicine.

The use of SHA*P* values in our study is particularly noteworthy. We did not just stop at model performance but delved deep into explainability, first for the global model and then for the cancer-specific models. This process allowed us to generate refined explainability that highlights not just which features are important, but how their importance varies across different types of cancer. The use of SHAP in both global and specific contexts is a novel approach that we believe adds substantial value to the study. While many studies, such as Maouche et al [52], use SHAP or similar methods, they often do so at a surface level, without the comprehensive, model-specific analysis that we provide. This depth of analysis is crucial for understanding the true implications of the model’s predictions and for making informed clinical decisions.

Finally, we conduct survival analysis after predicting cancer survival with explainable ML to translate model predictions into clinically meaningful insights about patient outcomes over time. While ML classifiers can distinguish between patients likely to survive or not, survival analysis provides a time-to-event perspective, capturing not just “if” but “when” an event such as death occurs. This temporal dimension allows researchers and clinicians to estimate hazard rates, median survival times, and differences between risk groups. Coupled with explainable ML (eg, SHA*P* values), survival analysis also helps validate whether the features driving predictions correspond to biologically and clinically relevant risk factors. Together, this integration strengthens both the predictive performance and interpretability of the model, supporting its potential use as a trustworthy decision-support tool in oncology practice.

### Limitations and Biases

We acknowledge several limitations that must be considered alongside the model’s strong performance. First, generalizability may be constrained by inherent biases in the MSK-MET dataset, which is a single-institution cohort from a tertiary care center. The sample underrepresents socioeconomically disadvantaged and racially diverse populations, with non-White ethnicities comprising less than 15%, and genomic profiling was preferentially performed in advanced-stage cases, potentially inflating the importance of features like TMB. These biases may partly explain the performance disparities observed across cancer types. Furthermore, technical limitations exist; for instance, linear and naive Bayes baselines may have been disadvantaged by the use of label encoding for categorical variables.

These factors underscore the necessity for several future steps before clinical deployment. External validation using diverse, multicenter cohorts with stratified sampling is crucial to improve equity, fairness, and generalizability. Prospective calibration should be rechecked under local prevalence shifts, with techniques like Platt scaling or isotonic regression applied, if necessary. Additionally, prospective benchmarking against oncologist-estimated prognoses and real-world deployment outcomes will be critical to establish clinical noninferiority or superiority. Finally, while this study provides a comprehensive classification and survival analysis, future work should focus on integrating these outputs; for example, by generating risk-stratified Kaplan-Meier curves at chosen thresholds to unify the decision framework across binary and time-to-event end points.

### Future Directions and Deployment Considerations

#### Improving Rare Cancer Predictions

Model performance was limited for rare cancers (eg, anal cancer: n=68; AUC=0.61), due to data scarcity. To address this, we suggest (1) using transfer learning to initialize models with global XGBoost weights, (2) using synthetic oversampling (eg, synthetic minority oversampling technique, adaptive synthetic sampling) during training, and (3) leveraging federated learning to aggregate data across institutions while preserving privacy. These techniques can bolster performance in underrepresented malignancies.

#### EHR Integration and Clinical Deployment

**Figure 6:**
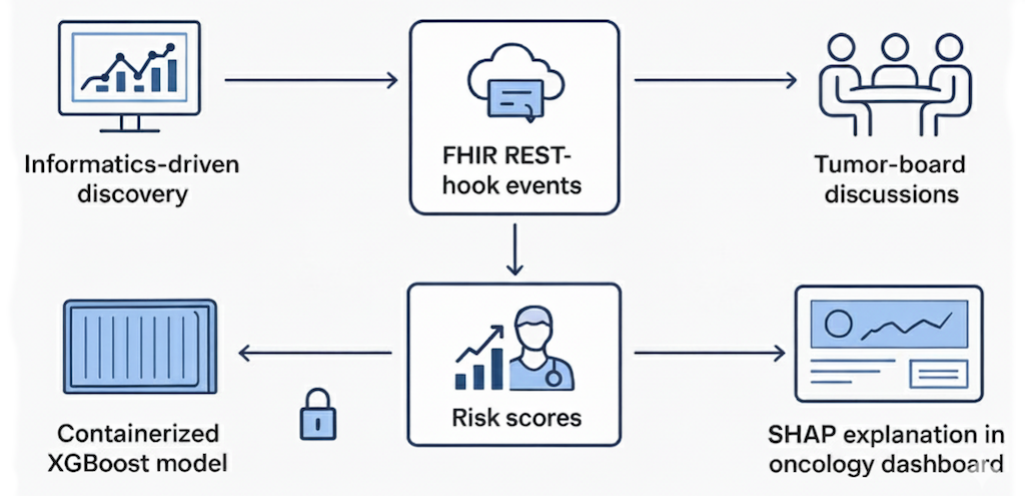
Electronic health record (EHR) integration and clinical deployment. Framework illustrating how patient risk scores and Shapley additive explanations (SHAP)–based explanations are integrated into the EHR and surfaced during tumor-board discussions to enable transparent, real-time clinical decision support. FHIR: Fast Healthcare Interoperability Resources; REST: Representational State Transfer; XGBoost: extreme gradient boosting.

While the primary aim of this study was informatics-driven discovery, the robustness of our model supports a clear pathway for clinical translation through EHR integration. We envision a real-time, standards-based system where automated risk alerts are seamlessly integrated into clinical workflows, particularly within tumor-board discussions (Figure 6). By leveraging Fast Healthcare Interoperability Resources Representational State Transfer hook events, patient risk scores could be dynamically updated and surfaced directly within the EHR as cases are reviewed. To ensure both privacy and computational efficiency, the XGBoost model would be deployed as a containerized service within the hospital’s secure analytics infrastructure (eg, Epic Cogito), keeping protected health information on premises while using available hardware acceleration.

A critical differentiator of this approach is the coupling of each risk alert with a SHAP-based explanation embedded directly into the oncology dashboard. This transforms the model from a “black box” into an “explain-and-act” tool, providing clinicians with immediate, interpretable rationale by highlighting the top clinical and genomic features contributing to an individual’s risk score. Alert protocols would be tiered and threshold-aware, directly translating the model’s predictive values into actionable clinical guidance. For instance, a low-risk classification (supported by an NPV of 79.8%) could justify lengthening follow-up intervals, while a high-risk flag (PPV of 70.3%) would prompt rapid biomarker reassessment and discussion of treatment intensification.

An initial rollout would prioritize safety and reliability through a prospective pilot study, continuous performance monitoring for model drift, and an implementation that minimally disrupts existing workflows. The validation evidence and explainable framework presented in this study provide a solid foundation for a potential regulatory submission as a Class II clinical decision support tool, paving the way for a new generation of transparent, AI-augmented oncology care.

### Conclusion

In this large-scale, pan-cancer study, we developed and validated an interpretable ML framework for predicting survivability in patients with metastatic cancer. By leveraging the comprehensive MSK-MET cohort, we demonstrated that an XGBoost classifier robustly predicts overall survival (AUC=0.82, accuracy=0.74), outperforming other conventional ML models. Crucially, the integration of SHAP explainability illuminated the model’s decision-making process, consistently identifying metastatic site count, TMB, fraction of genome altered, and the presence of liver and bone metastases as major prognostic features across diverse tumor types. This biological plausibility is fundamental for building clinical trust.

Our 2-tiered modeling approach, combining a unified pan-cancer perspective with targeted, cancer-specific submodels, provided both broad generalizability and nuanced, disease-tailored insights. This was evidenced by the varying performance across cancer types, such as the high predictive accuracy for prostate cancer (AUC=0.88) contrasted with the greater challenges in pancreatic cancer (AUC=0.68). Furthermore, the survival analysis corroborated the classification findings, with the XGBoost-Cox model (C-index=0.70) capturing nonlinear relationships more effectively than the traditional Cox model (C-index=0.66), and Kaplan-Meier curves starkly illustrating the significant survival disadvantage associated with metastatic disease.

The clinical use of our model is underscored by its strong predictive values (PPV=70% and NPV=80% at the Youden-optimal threshold) and its demonstrated net benefit across a range of decision thresholds. By reconciling high performance with transparent, actionable explanations, this work provides a foundational framework for the next generation of clinical decision-support tools in oncology. Future efforts should focus on external validation in multicenter cohorts, prospective evaluation integrated within EHR systems, and addressing performance gaps in rare cancer subtypes to ensure equitable and widespread clinical adoption.

## Appendix

Multimedia Appendix 1 Additional tables and figures.

## Abbreviations

AI: artificial intelligence
AUC: area under the curve
AUPRC: area under the precision-recall curve
AUROC: area under the receiver operating characteristic curve
C-index: concordance index
CPH: Cox proportional hazards
CV: cross validation
EHR: electronic health record
FDA: US Food and Drug Administration
FN: false negative
FP: false positive
MCC: Matthews correlation coefficient
ML: machine learning
MSK-MET: Memorial Sloan Kettering-Metastatic
NPV: negative predictive value
PPV: positive predictive value
ROC: receiver operating characteristic
SHAP: Shapley additive explanations
TMB: tumor mutational burden
TN: true negative
TP: true positive
XGBoost: extreme gradient boosting

## Data Availability

The datasets used in this study are available in the Memorial Sloan Kettering-Metastatic repository at cBioPortal [54]. The code is available at GitHub [55].

## References

[1] Siegel RL, Giaquinto AN, Jemal A (2024). Cancer statistics, 2024. CA Cancer J Clin.

[2] Li L, Xiong F, Wang Y, Zhang S, Gong Z, Li X, He Y, Shi L, Wang F, Liao Q, Xiang B, Zhou M, Li X, Li Y, Li G, Zeng Z, Xiong W, Guo C (2021). What are the applications of single-cell RNA sequencing in cancer research: a systematic review. J Exp Clin Cancer Res.

[3] Marusyk A, Polyak K (2010). Tumor heterogeneity: causes and consequences. Biochim Biophys Acta.

[4] Li L, Xiong F, Wang Y, Zhang S, Gong Z, Li X, He Y, Shi Lei, Wang Fuyan, Liao Qianjin, Xiang Bo, Zhou Ming, Li Xiaoling, Li Yong, Li Guiyuan, Zeng Zhaoyang, Xiong Wei, Guo Can (2021). What are the applications of single-cell RNA sequencing in cancer research: a systematic review. J Exp Clin Cancer Res.

[5] Esteva A, Kuprel B, Novoa RA, Ko J, Swetter SM, Blau HM, Thrun S (2017). Dermatologist-level classification of skin cancer with deep neural networks. Nature.

[6] Liu Y, Kim J, Balagurunathan Y, Hawkins S, Stringfield O, Schabath MB, Li Q, Qu F, Liu S, Garcia AL, Ye Z, Gillies RJ (2018). Prediction of pathological nodal involvement by CT-based radiomic features of the primary tumor in patients with clinically node-negative peripheral lung adenocarcinomas. Med Phys.

[7] Montazeri M, Montazeri M, Montazeri M, Beigzadeh A (2016). Machine learning models in breast cancer survival prediction. Technol Health Care.

[8] Zhou L, Wu X, Huang S, Wu G, Ye H, Wei Q, Bao L, Deng Y, Li X, Cui X, Dietrich CF (2020). Lymph node metastasis prediction from primary breast cancer US images using deep learning. Radiology.

[9] Foersch S, Eckstein M, Wagner DC, Gach F, Woerl A, Geiger J, Glasner C, Schelbert S, Schulz S, Porubsky S, Kreft A, Hartmann A, Agaimy A, Roth W (2021). Deep learning for diagnosis and survival prediction in soft tissue sarcoma. Ann Oncol.

[10] Doppalapudi S, Qiu RG, Badr Y (2021). Lung cancer survival period prediction and understanding: Deep learning approaches. Int J Med Inform.

[11] Zhang G, Shen X, Zhang YD, Luo Y, Luo J, Zhu D, Yang H, Wang W, Zhao B, Lu J (2022). Cross-modal prostate cancer segmentation via self-attention distillation. IEEE J Biomed Health Inform.

[12] Huang Y, Wei L, Hu Y, Shao N, Lin Y, He S, Shi H, Zhang X, Lin Y (2021). Multi-parametric MRI-based radiomics models for predicting molecular subtype and androgen receptor expression in breast cancer. Front Oncol.

[13] Chen T, Guestrin C (2016). XGBoost: a scalable tree boosting system. https://doi.org/10.1145/2939672.2939785.

[14] Liu Y, Wang Z, Cao X, Liu M, Zhong L (2025). Machine learning models for predicting survival in lung cancer patients undergoing microwave ablation. Front Med (Lausanne).

[15] Noman SM, Fadel YM, Henedak MT, Attia NA, Essam M, Elmaasarawii S, Fouad FA, Eltasawi EG, Al-Atabany W (2025). Leveraging survival analysis and machine learning for accurate prediction of breast cancer recurrence and metastasis. Sci Rep.

[16] Buk Cardoso L, Cunha Parro V, Verzinhasse Peres S, Curado MP, Fernandes GA, Wünsch Filho V, Natasha Toporcov T (2023). Machine learning for predicting survival of colorectal cancer patients. Sci Rep.

[17] Ben Kridis W, Wang Z, Guven DC, Dharmarajan A (2024). Editorial: advances in the treatment of hormonal receptor positive (HR+) breast cancer. Front Oncol.

[18] Breiman L (2001). Random Forests. Mach Learn.

[19] Cortes C, Vapnik V (1995). Support-vector networks. Mach Learn.

[20] LeCun Y, Bengio Y, Hinton G (2015). Deep learning. Nature.

[21] Liu Z, Liu L, Weng S, Guo C, Dang Q, Xu H, Wang L, Lu T, Zhang Y, Sun Z, Han X (2022). Machine learning-based integration develops an immune-derived lncRNA signature for improving outcomes in colorectal cancer. Nat Commun.

[22] He B, Bergenstråhle L, Stenbeck L, Abid A, Andersson A, Borg Å, Maaskola J, Lundeberg J, Zou J (2020). Integrating spatial gene expression and breast tumour morphology via deep learning. Nat Biomed Eng.

[23] Huang S, Cai N, Pacheco PP, Narrandes S, Wang Y, Xu W (2018). Applications of support vector machine (SVM) learning in cancer genomics. Cancer Genomics Proteomics.

[24] Nguyen TB, Do DN, Nguyen-Thi M, Hoang-The H, Tran T, Nguyen-Thanh T (2022). Identification of potential crucial genes and key pathways shared in inflammatory bowel disease and cervical cancer by machine learning and integrated bioinformatics. Comput Biol Med.

[25] Stefanou IK, Dovrolis N, Gazouli M, Theodorou D, Zografos GK, Toutouzas KG (2022). miRNAs expression pattern and machine learning models elucidate risk for gastric GIST. Cancer Biomark.

[26] Pawelka D, Laczmanska I, Karpinski P, Supplitt S, Witkiewicz W, Knychalski B, Pelak J, Zebrowska P, Laczmanski L (2022). Machine-learning-based analysis identifies miRNA expression profile for diagnosis and prediction of colorectal cancer: a preliminary study. Cancer Genomics Proteomics.

[27] Ghobadi MZ, Emamzadeh R, Afsaneh E (2022). Exploration of mRNAs and miRNA classifiers for various ATLL cancer subtypes using machine learning. BMC Cancer.

[28] Ding W, Chen G, Shi T (2019). Integrative analysis identifies potential DNA methylation biomarkers for pan-cancer diagnosis and prognosis. Epigenetics.

[29] Liang N, Li B, Jia Z, Wang C, Wu P, Zheng T, Wang Y, Qiu F, Wu Y, Su J, Xu J, Xu F, Chu H, Fang S, Yang X, Wu C, Cao Z, Cao L, Bing Z, Liu H, Li L, Huang C, Qin Y, Cui Y, Han-Zhang H, Xiang J, Liu H, Guo X, Li S, Zhao H, Zhang Z (2021). Ultrasensitive detection of circulating tumour DNA via deep methylation sequencing aided by machine learning. Nat Biomed Eng.

[30] Avanzo M, Stancanello J, Pirrone G, Sartor G (2020). Radiomics and deep learning in lung cancer. Strahlenther Onkol.

[31] Janssen BV, Verhoef S, Wesdorp NJ, Huiskens J, de Boer OJ, Marquering H, Stoker J, Kazemier G, Besselink MG (2022). Imaging-based machine-learning models to predict clinical outcomes and identify biomarkers in pancreatic cancer: a scoping review. Ann Surg.

[32] Stanzione A, Verde F, Romeo V, Boccadifuoco F, Mainenti PP, Maurea S (2021). Radiomics and machine learning applications in rectal cancer: current update and future perspectives. World J Gastroenterol.

[33] Wang R, Dai W, Gong J, Huang M, Hu T, Li H, Lin K, Tan C, Hu H, Tong T, Cai G (2022). Development of a novel combined nomogram model integrating deep learning-pathomics, radiomics and immunoscore to predict postoperative outcome of colorectal cancer lung metastasis patients. J Hematol Oncol.

[34] Nguyen B, Fong C, Luthra A, Smith SA, DiNatale RG, Nandakumar S, Walch H, Chatila WK, Madupuri R, Kundra R, Bielski CM, Mastrogiacomo B, Donoghue MTA, Boire A, Chandarlapaty S, Ganesh K, Harding JJ, Iacobuzio-Donahue CA, Razavi P, Reznik E, Rudin CM, Zamarin D, Abida W, Abou-Alfa GK, Aghajanian C, Cercek A, Chi P, Feldman D, Ho AL, Iyer G, Janjigian YY, Morris M, Motzer RJ, O'Reilly EM, Postow MA, Raj NP, Riely GJ, Robson ME, Rosenberg JE, Safonov A, Shoushtari AN, Tap W, Teo MY, Varghese AM, Voss M, Yaeger R, Zauderer MG, Abu-Rustum N, Garcia-Aguilar J, Bochner B, Hakimi A, Jarnagin WR, Jones DR, Molena D, Morris L, Rios-Doria E, Russo P, Singer S, Strong VE, Chakravarty D, Ellenson LH, Gopalan A, Reis-Filho JS, Weigelt B, Ladanyi M, Gonen M, Shah SP, Massague J, Gao J, Zehir A, Berger MF, Solit DB, Bakhoum SF, Sanchez-Vega F, Schultz N (2022). Genomic characterization of metastatic patterns from prospective clinical sequencing of 25,000 patients. Cell.

[35] Ramírez-Mena A, Andrés-León E, Alvarez-Cubero MJ, Anguita-Ruiz A, Martinez-Gonzalez LJ, Alcala-Fdez J (2023). Explainable artificial intelligence to predict and identify prostate cancer tissue by gene expression. Comput Methods Programs Biomed.

[36] Mandrekar JN (2010). Receiver operating characteristic curve in diagnostic test assessment. J Thorac Oncol.

[37] Nahm FS (2022). Receiver operating characteristic curve: overview and practical use for clinicians. Korean J Anesthesiol.

[38] Philip MM, Welch A, McKiddie F, Nath M (2023). A systematic review and meta-analysis of predictive and prognostic models for outcome prediction using positron emission tomography radiomics in head and neck squamous cell carcinoma patients. Cancer Med.

[39] Dretzke J, Abou-Foul AK, Albon E, Hillier B, Scandrett K, Price MJ, Moore DJ, Mehanna H, Nankivell P (2024). Systematic review of prognostic models for predicting recurrence and survival in patients with treated oropharyngeal cancer. BMJ Open.

[40] Gouthamchand V, Fonseca LAF, Hoebers FJP, Fijten R, Dekker A, Wee L (2025). Prognostic modeling in headneck cancer: deep learning or handcrafted radiomics?. BJR Artif Intell.

[41] Joshi G, Jain A, Araveeti SR, Adhikari S, Garg H, Bhandari M (2024). FDA-approved artificial intelligence and machine learning (AI/ML)-Enabled medical devices: an updated landscape. Electronics.

[42] (2025). Artificial intelligence and machine learning (AI/ML)-enabled medical devices. US Food and Drug Administration.

[43] Fawcett T (2006). An introduction to ROC analysis. Pattern Recognit Lett.

[44] Wolpert DH (1992). Stacked generalization. Neural Netw.

[45] Kuncheva L, Whitaker C (2003). Measures of diversity in classifier ensembles and their relationship with the ensemble accuracy. Mach Learn.

[46] Davoli T, Uno H, Wooten EC, Elledge SJ (2017). Tumor aneuploidy correlates with markers of immune evasion and with reduced response to immunotherapy. Science.

[47] Tsimberidou A (2015). Targeted therapy in cancer. Cancer Chemother Pharmacol.

[48] Zhao B, Gabriel RA, Vaida F, Lopez NE, Eisenstein S, Clary BM (2020). Predicting overall survival in patients with metastatic rectal cancer: a machine learning approach. J Gastrointest Surg.

[49] Tapak L, Shirmohammadi-Khorram N, Amini P, Alafchi B, Hamidi O, Poorolajal J (2019). Prediction of survival and metastasis in breast cancer patients using machine learning classifiers. Clin Epidemiol Glob Health.

[50] Nicolò C, Périer C, Prague M, Bellera C, MacGrogan G, Saut O, Benzekry S (2020). Machine learning and mechanistic modeling for prediction of metastatic relapse in early-stage breast cancer. JCO Clin Cancer Inform.

[51] Kourou K, Exarchos TP, Exarchos KP, Karamouzis MV, Fotiadis DI (2015). Machine learning applications in cancer prognosis and prediction. Comput Struct Biotechnol J.

[52] Maouche I, Terrissa LS, Benmohammed K, Zerhouni N (2023). An explainable AI approach for breast cancer metastasis prediction based on clinicopathological data. IEEE Trans Biomed Eng.

[53] Baldin I, Nikolich A, Griffioen J, Monga Iis, Wang K, Lehman T, Ruth P (2019). FABRIC: a national-scale programmable experimental network infrastructure. IEEE Internet Comput.

[54] cBioPortal.

[55] MU-Data-Science/GAF. GitHub.

