# Supplementary material for: Explainable AI for Predicting Mortality Risk in Metastatic Cancer: Retrospective Cohort Study Using the Memorial Sloan Kettering-Metastatic Dataset
Source: JMIR Cancer. 2026 Jan 13;12:e74196. doi: 10.2196/74196 (PMC12848487; doi:10.2196/74196)
Supplement: Multimedia Appendix 1 [file cancer_v12i1e74196_app1.docx]

**EXPLAINABLE ARTIFICIAL INTELLIGENCE FOR PREDICTING MORTALITY RISK IN METASTATIC CANCER: RETROSPECTIVE COHORT STUDY USING THE MSK-MET DATASET**

**SUPPLEMENTAL INFORMATION:**

**Supplementary images for Figure 2**


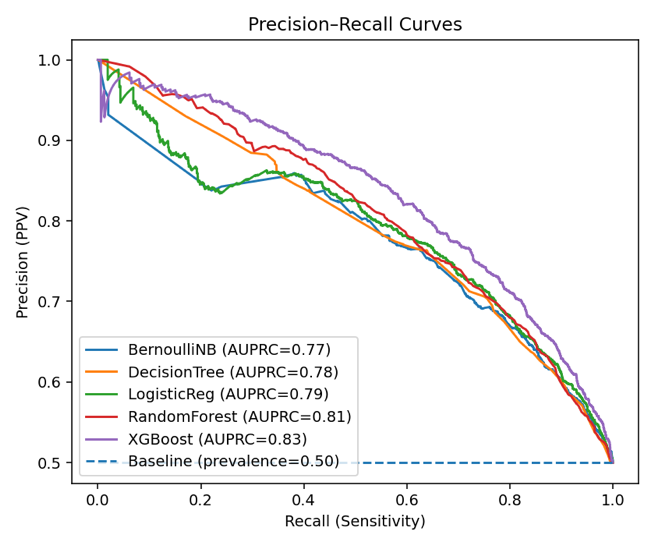
(A)


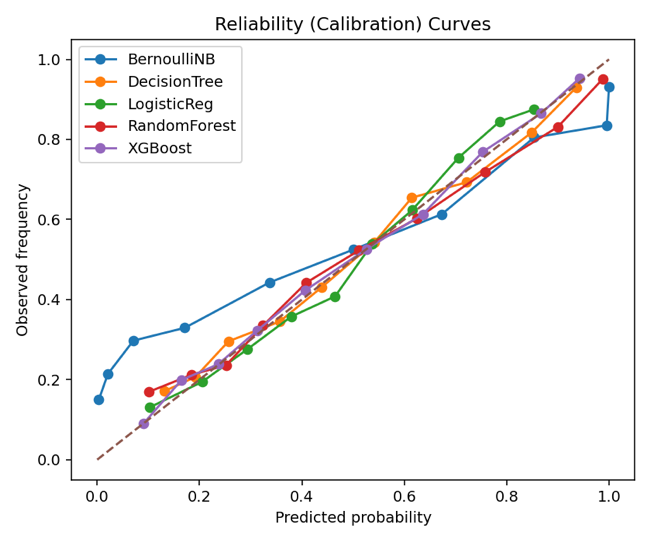
(B)
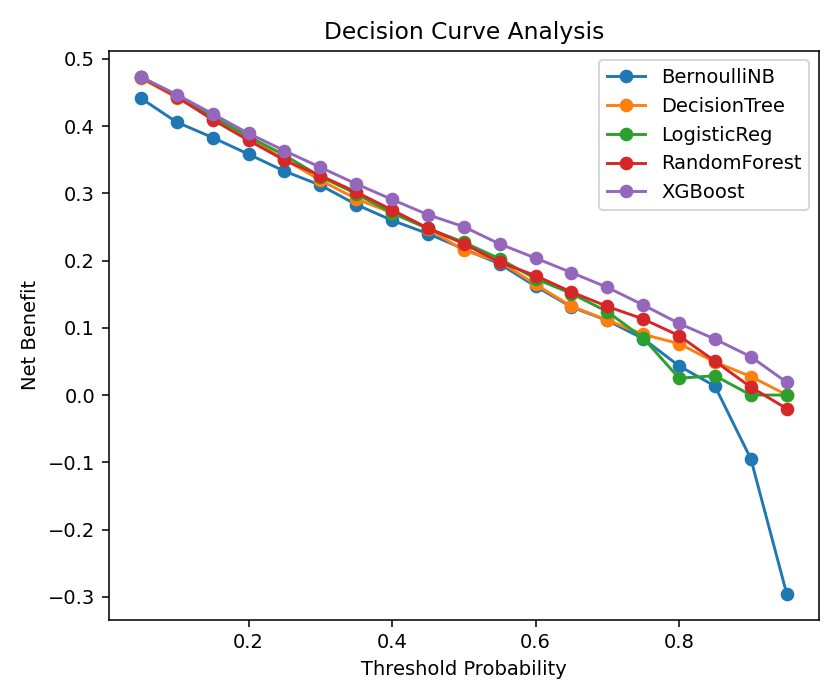
(C)


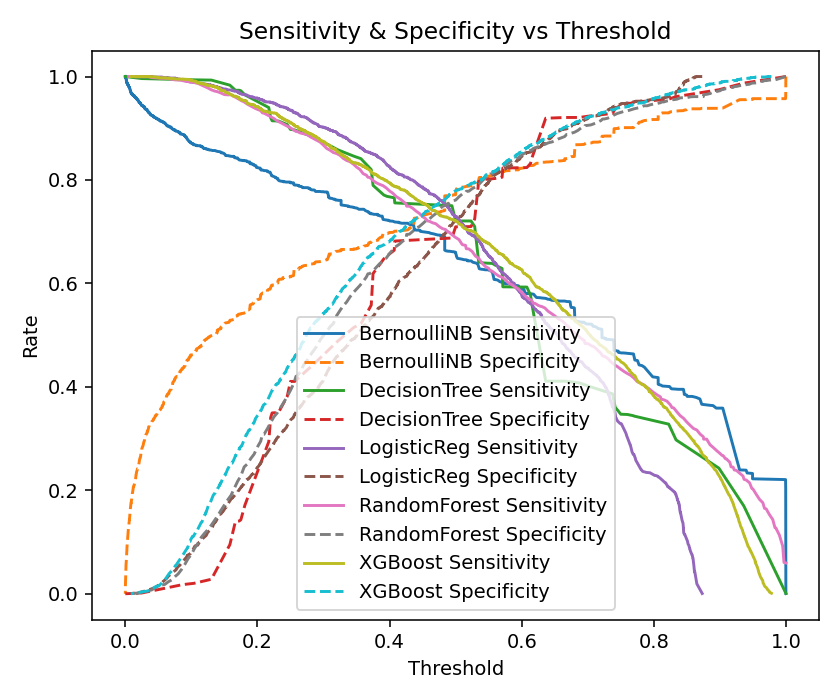
(D)

**Figure S2.** Discrimination, calibration, clinical utility, and threshold trade-offs across tuned classifiers on the held-out test set. Five models (BernoulliNB, Decision Tree, Logistic Regression, Random Forest, XGBoost) were tuned via 5-fold stratified GridSearchCV (AUC scoring) and evaluated on a stratified 20% test split at the original prevalence. (A) Precision-Recall curves with AUPRC; the dashed line marks the baseline precision (prevalence). (B) Reliability (calibration) curves from 10 quantile bins; the diagonal indicates perfect calibration. (C) Decision-curve analysis (net benefit vs threshold probability). (D) Sensitivity and specificity as a function of decision threshold. Higher is better for AUC/AUPRC, calibration closeness to the diagonal, and net benefit.

**Supplementary table for Table 2**

*Table S2. Scalar performance on the held-out test set with 95% bootstrap confidence intervals. For each tuned model we report AUC, AUPRC, and Brier score (lower is better), with 95% CIs estimated via 1,000-sample stratified bootstrap. Test-set prevalence is shown to contextualize AUPRC and PPV/NPV.*

| **Model** | **AUC** | **AUC CI_low** | **AUC CI_high** | **AUPRC** | **AUPRC CI_low** | **AUPRC CI_high** | **Brier** | **Brier CI_low** | **Brier CI_high** | **Prevalence** |
| --- | --- | --- | --- | --- | --- | --- | --- | --- | --- | --- |
| XGBoost: learning_rate=0.01, max_depth=5, n_estimators=500 | 0.82 | 0.81 | 0.84 | 0.83 | 0.81 | 0.85 | 0.17 | 0.16 | 0.18 | 0.5 |
| Random Forest: n_estimators=200, max_features=auto | 0.80 | 0.78 | 0.81 | 0.81 | 0.79 | 0.82 | 0.18 | 0.18 | 0.19 | 0.5 |
| Logistic Regression: C=10, penalty=l2, solver=liblinear | 0.79 | 0.78 | 0.81 | 0.79 | 0.77 | 0.81 | 0.19 | 0.18 | 0.19 | 0.5 |
| Decision Tree: criterion=gini, max depth= 8, min_samples_leaf= 1,  min_samples_split= 10 | 0.78 | 0.77 | 0.80 | 0.78 | 0.76 | 0.80 | 0.19 | 0.18 | 0.20 | 0.5 |
| Naïve Bayes: α = 10.0, binarize= 0.5, fit_prior=False | 0.78 | 0.77 | 0.80 | 0.77 | 0.75 | 0.79 | 0.21 | 0.20 | 0.22 | 0.5 |

**Supplementary table for Table 3**

**Table S3.** Confusion-matrix-derived metrics at pre-specified operating points. For each tuned model we report TP, FP, TN, FN and derived metrics sensitivity (recall), specificity, precision, PPV, NPV, F1, balanced accuracy, Matthews correlation coefficient (MCC), Cohen’s κ, and accuracy at three thresholds: a fixed 0.50 cut-point, the Youden-J optimum (maximizing TPR-FPR), and a clinically constrained operating point targeting sensitivity ≥0.85 (smallest threshold achieving the target). Metrics are computed on the untouched test set at original prevalence with 1,000-sample bootstrap CIs for scalar summaries.

| **Model** | **Operating point** | **Threshold** | **TP** | **FP** | **TN** | **FN** | **Sensitivity** | **Specificity** | **Precision** | **NPV** | **F1** | **Balanced Acc** | **MCC** | **κ** | **Accuracy** |
| --- | --- | --- | --- | --- | --- | --- | --- | --- | --- | --- | --- | --- | --- | --- | --- |
| Naïve Bayes | 0.5 | 0.50 | 1338 | 454 | 1580 | 696 | 0.66 | 0.78 | 0.75 | 0.69 | 0.70 | 0.72 | 0.44 | 0.43 | 0.72 |
|  | YoudenJ | 0.50 | 1329 | 442 | 1592 | 705 | 0.65 | 0.78 | 0.75 | 0.69 | 0.70 | 0.72 | 0.44 | 0.44 | 0.72 |
|  | sens>=0.85 | 0.15 | 1729 | 971 | 1063 | 305 | 0.85 | 0.52 | 0.64 | 0.78 | 0.73 | 0.69 | 0.39 | 0.37 | 0.69 |
| Decision Tree | 0.5 | 0.50 | 1466 | 591 | 1443 | 568 | 0.72 | 0.71 | 0.71 | 0.72 | 0.72 | 0.72 | 0.43 | 0.43 | 0.72 |
|  | YoudenJ | 0.56 | 1299 | 402 | 1632 | 735 | 0.64 | 0.80 | 0.76 | 0.69 | 0.70 | 0.72 | 0.45 | 0.44 | 0.72 |
|  | sens>=0.85 | 0.32 | 1746 | 1041 | 993 | 288 | 0.86 | 0.49 | 0.63 | 0.78 | 0.72 | 0.67 | 0.37 | 0.35 | 0.67 |
| Logistic Regression | 0.5 | 0.50 | 1483 | 559 | 1475 | 551 | 0.73 | 0.73 | 0.73 | 0.73 | 0.73 | 0.73 | 0.45 | 0.45 | 0.73 |
|  | YoudenJ | 0.53 | 1399 | 471 | 1563 | 635 | 0.69 | 0.77 | 0.75 | 0.71 | 0.72 | 0.73 | 0.46 | 0.46 | 0.73 |
|  | sens>=0.85 | 0.38 | 1729 | 940 | 1094 | 305 | 0.85 | 0.54 | 0.65 | 0.78 | 0.74 | 0.69 | 0.41 | 0.39 | 0.69 |
| Random Forest | 0.5 | 0.50 | 1401 | 486 | 1548 | 633 | 0.69 | 0.76 | 0.74 | 0.71 | 0.71 | 0.72 | 0.45 | 0.45 | 0.72 |
|  | YoudenJ | 0.49 | 1432 | 506 | 1528 | 602 | 0.70 | 0.75 | 0.74 | 0.72 | 0.72 | 0.73 | 0.46 | 0.46 | 0.73 |
|  | sens>=0.85 | 0.32 | 1731 | 940 | 1094 | 303 | 0.85 | 0.54 | 0.65 | 0.78 | 0.74 | 0.69 | 0.41 | 0.39 | 0.69 |
| XGBoost | 0.5 | 0.50 | 1466 | 448 | 1586 | 568 | 0.72 | 0.78 | 0.77 | 0.74 | 0.74 | 0.75 | 0.50 | 0.50 | 0.75 |
|  | YoudenJ | 0.50 | 1466 | 447 | 1587 | 568 | 0.72 | 0.78 | 0.77 | 0.74 | 0.74 | 0.75 | 0.50 | 0.50 | 0.75 |
|  | sens>=0.85 | 0.33 | 1731 | 846 | 1188 | 303 | 0.85 | 0.58 | 0.67 | 0.80 | 0.75 | 0.72 | 0.45 | 0.44 | 0.72 |

**Supplementary Table 6**

**Table S6.** Top 10 SHAP-ranked features (Rank 1 = most important) for the global XGBoost mortality classifier and each cancer-specific XGBoost model (non-small cell lung, colorectal, breast, pancreatic, and prostate cancer). The table highlights substantial overlap in influential predictors across models, with metastatic site count, distant metastases to liver and bone, tumor mutation burden, fraction of genome altered, MSI score, sample type/coverage, and select organ-specific metastatic sites repeatedly emerging as key drivers of predicted mortality risk.

| **Rank** | **Global XGBoost** | **Non-Small Cell Lung Cancer** | **Colorectal Cancer** | **Breast Cancer** | **Pancreatic Cancer** | **Prostate Cancer** |
| --- | --- | --- | --- | --- | --- | --- |
| **1** | Met Site Count | Met Site Count | Met Count | Met Site Count | Met Site Count | Met Count |
| **2** | Gene Panel | Gene Panel | Distant Mets: Liver | Met Count | Distant Mets: Liver | Distant Mets: Bone |
| **3** | Distant Mets: Liver | Met Count | Gene Panel | Distant Mets: Liver | Gene Panel | Distant Mets: Liver |
| **4** | Met Count | Distant Mets: Bone | Distant Mets: Intra-Abdominal | Gene Panel | Sample coverage | MSI Score |
| **5** | Cancer Type | MSI Score | Met Site Count | Sample Type | Oncotree Code | Distant Mets: Male Genital |
| **6** | Distant Mets: Bone | TMB (nonsynonymous) | Distant Mets: Unspecified | Subtype | Distant Mets: Intra-Abdominal | Fraction of genome altered |
| **7** | TMB (nonsynonymous) | Sex | MSI Score | Metastatic patient | MSI Score | Sample Type |
| **8** | Sample Type | Sample Type | TMB (nonsynonymous) | MSI Score | Fraction of genome altered | Sample coverage |
| **9** | Subtype | Distant Mets: Liver | Sample coverage | Distant Mets: Unspecified | TMB (nonsynonymous) | TMB (nonsynonymous) |
| **10** | Distant Mets: Intra-Abdominal | Sample coverage | Fraction of genome altered | Fraction of genome altered | Race Category | Gene Panel |

**Supplementary images for Figure 3**


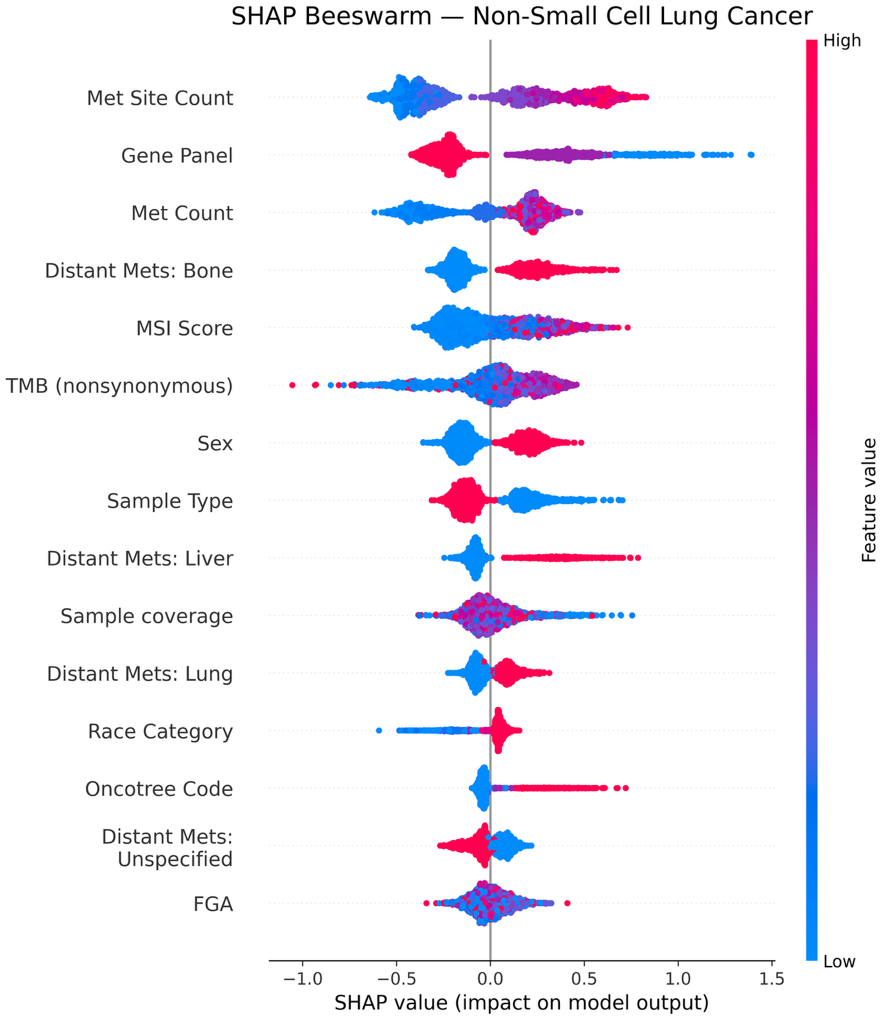


(A)


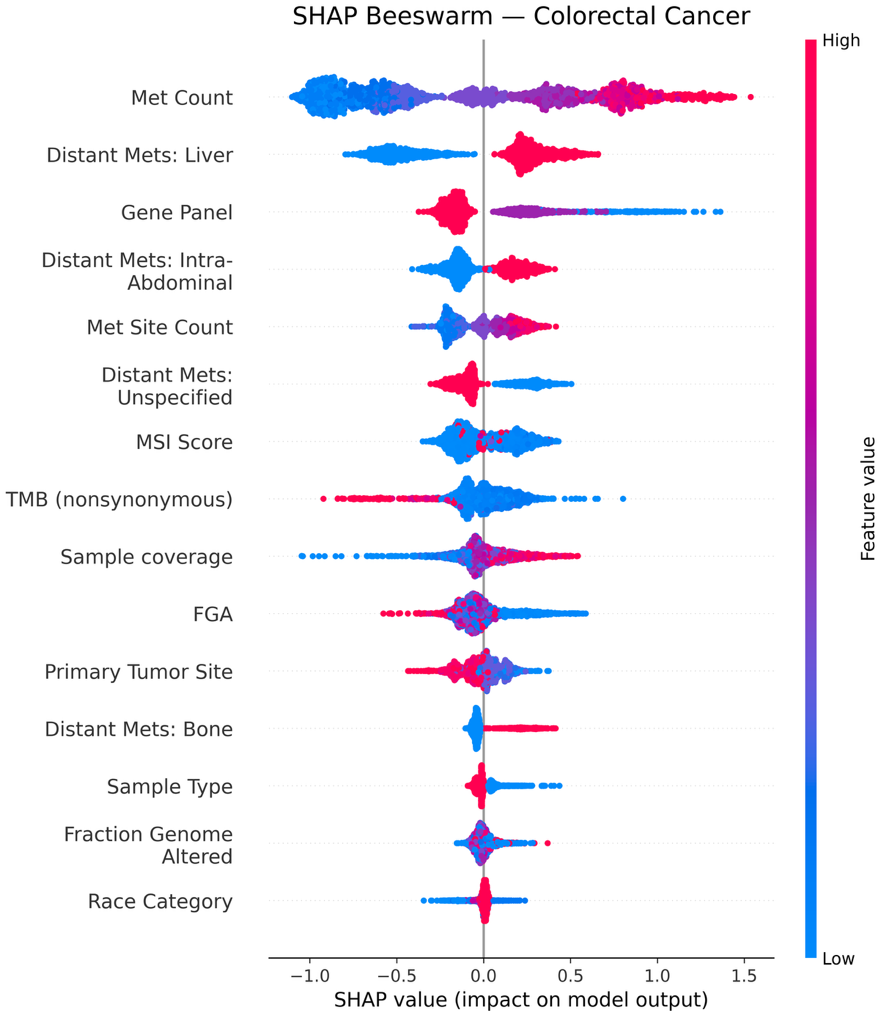
 (B)


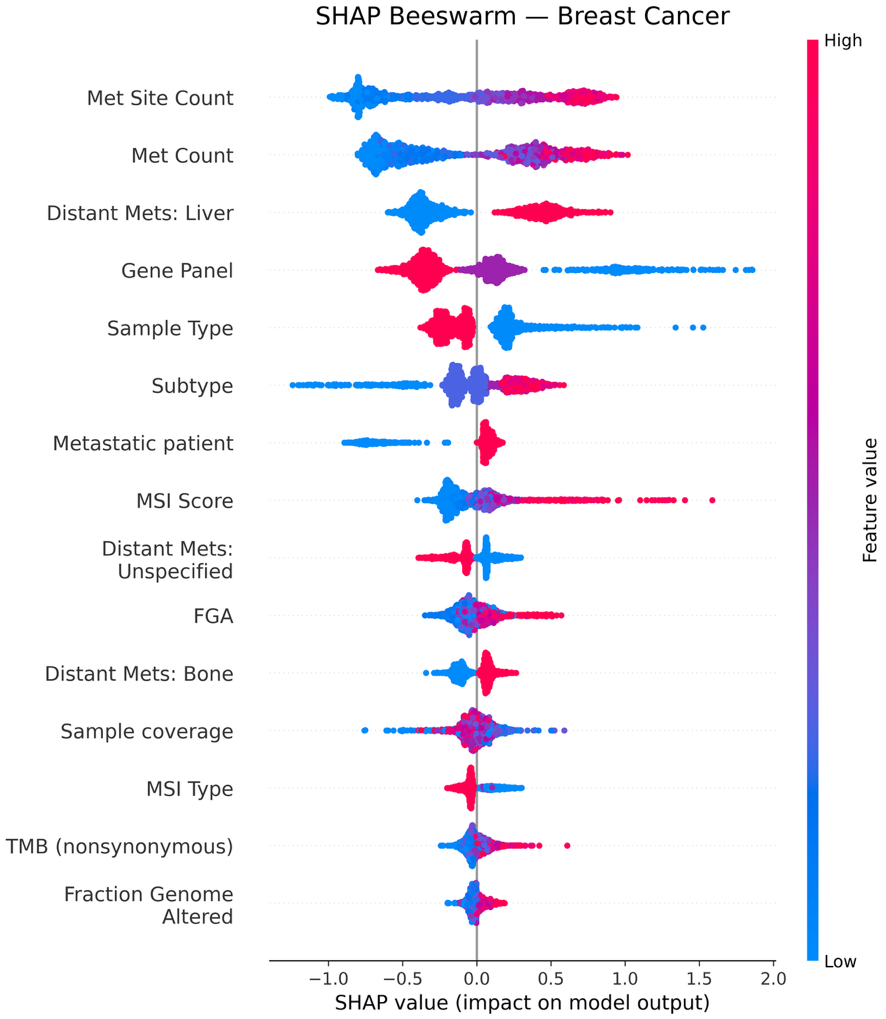
 (C)


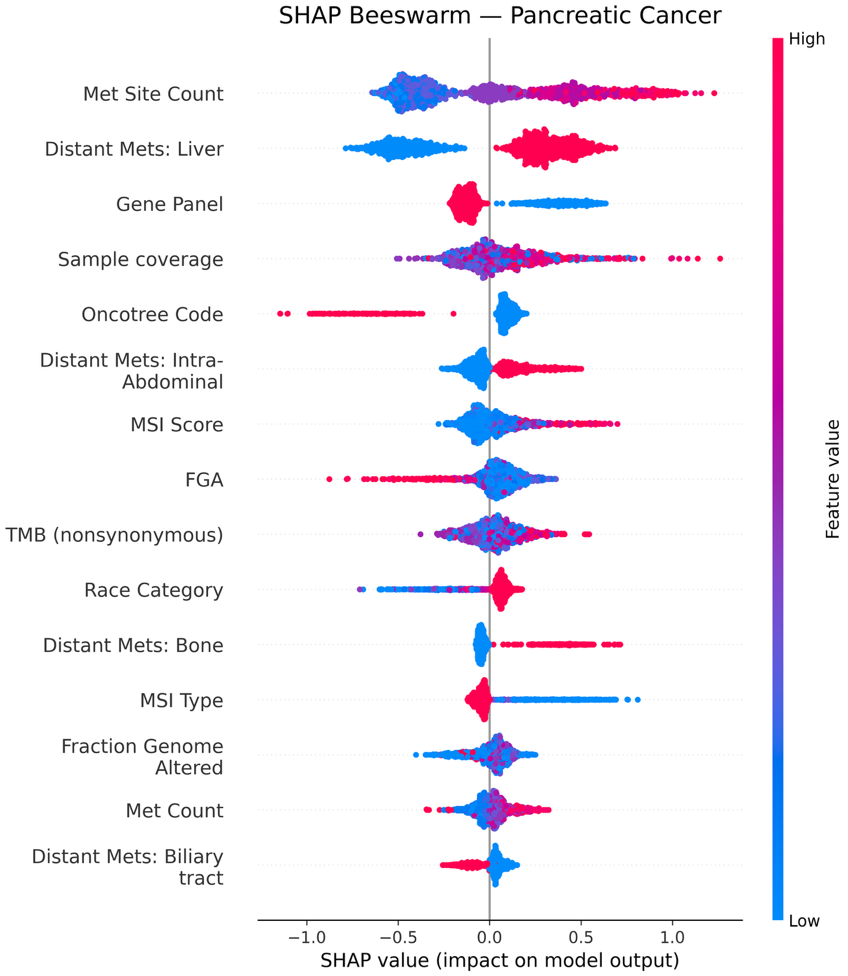


(D)


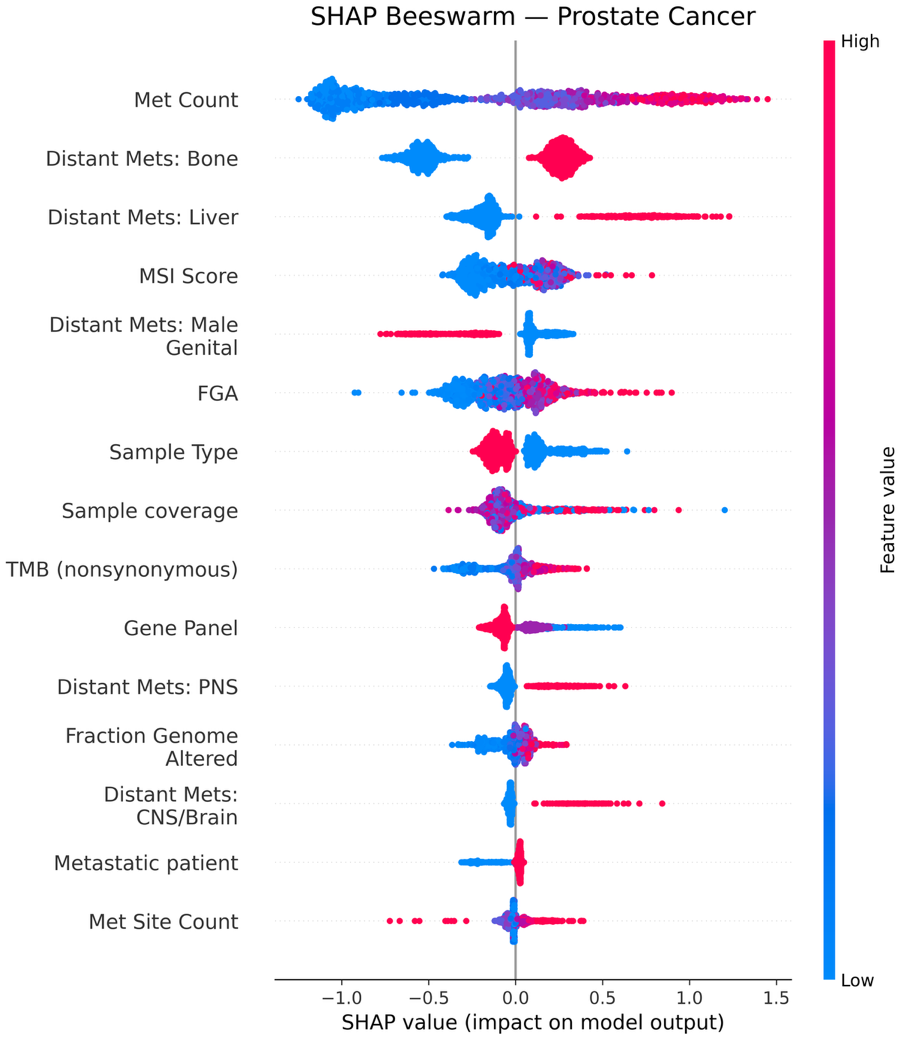


(E)

**Figure S3.** Cancer-specific SHAP beeswarm plots for XGBoost mortality classifiers in the five largest primary tumor types. Panels A-E correspond to non-small cell lung, colorectal, breast, pancreatic, and prostate cancer, respectively. Within each panel, features are ordered by mean absolute SHAP value, and each point represents an individual patient, with horizontal position indicating the direction and magnitude of impact on predicted mortality risk and color denoting low to high feature values. The plots highlight both shared drivers of mortality risk such as metastatic site count, distant metastases to liver and bone, tumor mutation burden, fraction of genome altered, and MSI score and organ-specific patterns that modulate model predictions across cancer types.

**Supplementary images for Figure 5**


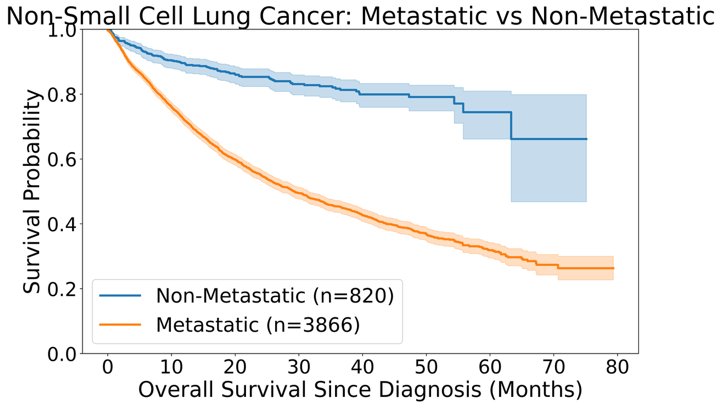
(A)


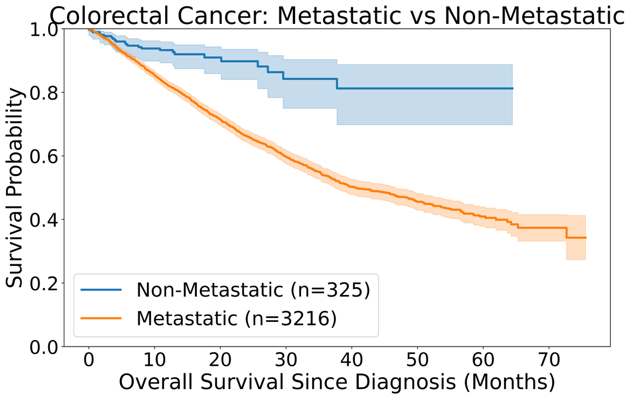
(B) **
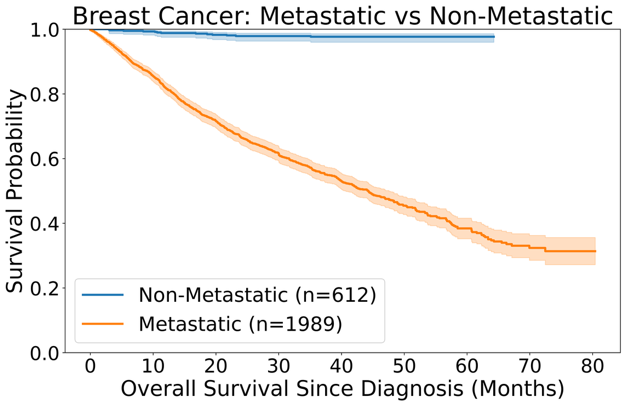
** (C)


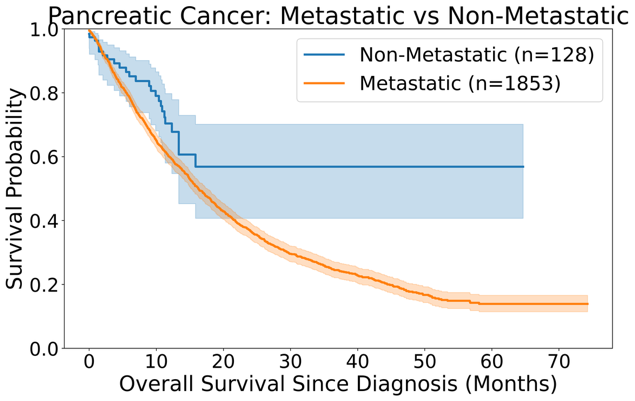
(D)
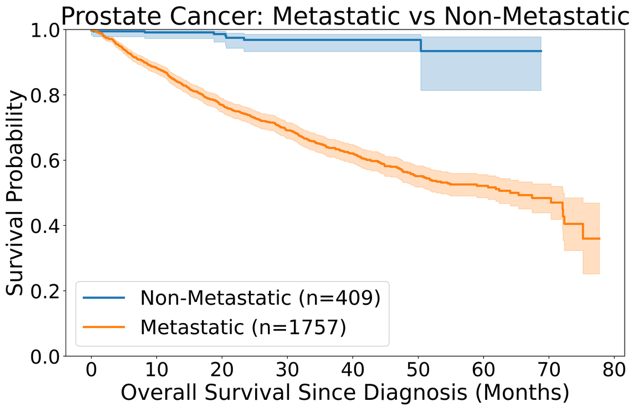
(E)

**Figure S5.** Kaplan-Meier overall survival curves comparing metastatic and non-metastatic patients in the full MSK-MET cohort and the five largest primary cancer types. Panel A shows pan-cancer survival stratified by metastatic status across all subgroups, while panels B-F correspond to non–small cell lung, colorectal, breast, pancreatic, and prostate cancer, respectively. Across all panels, patients with metastases experience steeper declines in survival probability and shorter median survival times than their non-metastatic counterparts, reinforcing the profound adverse impact of metastatic spread on prognosis across diverse tumor types.

**Supplementary Table 7**

**Table S7.** Important Features of the XGBoost Survival Analysis.

| **Feature** | **Importance** |
| --- | --- |
| Distant Mets: Liver | 0.315575 |
| Fraction of Genome Altered | 0.155434 |
| TMB (nonsynonymous) | 0.138214 |
| Met Site Count | 0.103948 |
| Metastatic patient | 0.102230 |
| Distant Mets: Bone | 0.092516 |
| Distant Mets: Lung | 0.048726 |
| Sample Type | 0.043358 |

**SupplementaryTable 8**

**Table S8.** Comparison of our paper and recent papers related to predicting survival of patients with metastatic cancer. GM and LM stand for Global model and Local model (Cancer-specific model), respectively.

| **Paper** | **XGBoost** | **Naïve**  **Bayes** | **Decision**  **Tree** | **Logistic**  **Regression** | **Random**  **Forest** | **GM/LM** | **SHAP**  **Analysis** | **Survival**  **Analysis** |
| --- | --- | --- | --- | --- | --- | --- | --- | --- |
| Zhao et al [[48](#zhao2020_mrc_survival_ml)] | *×* | *×* | *×* | *×* | *×* | *×* | *×* | *×* |
| Tapak et al [[49](#tapak2019_breast_survival_ml)] | *×* | ✓ | *×* | *×* | ✓ | *×* | *×* | *×* |
| Nicolò et al [[50](#nicolo2020_breast_relapse_ml)] | *×* | *×* | *×* | *×* | ✓ | *×* | *×* | *×* |
| Kourou et al [[51](#kourou2015_cancer_prognosis_ml)] | *×* | *×* | ✓ | *×* | *×* | *×* | *×* | *×* |
| Maouche et al [[52](#maouche2023_breast_metastasis_xai)] | ✓ | *×* | ✓ | ✓ | *×* | *×* | ✓ | *×* |
| **Our Paper** | ✓ | ✓ | ✓ | ✓ | ✓ | ✓ | ✓ | ✓ |
